# Enhanced Membrane Fluidization and Cholesterol Displacement by 1-Heptanol Inhibit Mast Cell Effector Functions

**DOI:** 10.3390/cells12162069

**Published:** 2023-08-15

**Authors:** Viktor Bugajev, Lubica Draberova, Pavol Utekal, Michaela Blazikova, Magda Tumova, Petr Draber

**Affiliations:** 1 Laboratory of Signal Transduction, Institute of Molecular Genetics of the Czech Academy of Sciences, 14220 Prague, Czech Republic; draberlu@img.cas.cz (L.D.); pavol.utekal@img.cas.cz (P.U.); magda.tumova@img.cas.cz (M.T.); 2 Light Microscopy Core Facility, Institute of Molecular Genetics of the Czech Academy of Sciences, 14220 Prague, Czech Republic; michaela.blazikova@img.cas.cz

**Keywords:** alkanol, membrane fluidizer, heat shock response, STIM1-ORAI1 coupling, store-operated calcium entry, FcεRI signaling, flow-FRET, FRAP

## Abstract

Signal transduction by the high-affinity IgE receptor (FcεRI) depends on membrane lipid and protein compartmentalization. Recently published data show that cells treated with 1-heptanol, a cell membrane fluidizer, exhibit changes in membrane properties. However, the functional consequences of 1-heptanol-induced changes on mast cell signaling are unknown. This study shows that short-term exposure to 1-heptanol reduces membrane thermal stability and dysregulates mast cell signaling at multiple levels. Cells treated with 1-heptanol exhibited increased lateral mobility and decreased internalization of the FcεRI. However, this did not affect the initial phosphorylation of the FcεRI-β chain and components of the SYK/LAT1/PLCγ1 signaling pathway after antigen activation. In contrast, 1-heptanol inhibited SAPK/JNK phosphorylation and effector functions such as calcium response, degranulation, and cytokine production. Membrane hyperfluidization induced a heat shock-like response via increased expression of the heat shock protein 70, increased lateral diffusion of ORAI1-mCherry, and unsatisfactory performance of STIM1-ORAI1 coupling, as determined by flow-FRET. Furthermore, 1-heptanol inhibited the antigen-induced production of reactive oxygen species and potentiated stress-induced plasma membrane permeability by interfering with heat shock protein 70 activity. The combined data suggest that 1-heptanol-mediated membrane fluidization does not interfere with the earliest biochemical steps of FcεRI signaling, such as phosphorylation of the FcεRI-β chain and components of the SYK/LAT/PLCγ1 signaling pathway, instead inhibiting the FcεRI internalization and mast cell effector functions, including degranulation and cytokine production.

## 1. Introduction

Plasma membrane lipids are organized into liquid-disordered (Ld)-like regions and liquid-ordered (Lo)-like domains, which are thought to be present in native cells as nanoscale domains [1,2]. The Lo-like domains, rich in cholesterol and sphingolipids, are preferentially occupied by proteins with a glycosylphosphatidylinositol (GPI) anchor or by acyl groups (e.g., myristoyl and palmitoyl). Several models have been coined to describe the initial steps of high-affinity receptor for IgE (FcεRI) and B cell receptor signaling at the plasma membrane [3,4]. The prevailing hypothesis assumes that in resting mast cells and basophils, FcεRI is present mainly in Ld-like regions; upon antigen-mediated crosslinking of IgE-sensitized FcεRI, it associates with Lo-like domains, where FcεRI-β and -γ chains are accessible for phosphorylation by LYN [3,5]. Recently, it has been shown that the aggregated FcεRI stabilizes surrounding Lo-like nanodomains through synergistic lipid-based, protein-based, and steric interactions, causing preferential exclusion of protein tyrosine phosphatase α and initiation of LYN-mediated phosphorylation [2]. It should be noted that the role of reactive oxygen species (ROS), which inactivate ROS-sensitive protein tyrosine phosphatases in the initial step by oxidation of their cysteine residues, is being considered, as these phosphatases and LYN are simultaneously found in the resting state complex with FcεRI [6,7].

Cholesterol has a critical role in the properties of the Lo-like domains. This small lipid molecule maintains membrane structural integrity and regulates cell membrane fluidity [8,9,10]. Studies with methyl-β-cyclodextrin (MβCD), a widely used drug to extract cholesterol from the outer plasma membrane leaflet [9], showed that MβCD treatment caused a decrease in the phosphorylation of FcεRI-β and -γ chains in antigen-activated rat basophil leukemia (RBL)-2H3 cells without affecting degranulation. Notably, the phosphorylation of FcεRI-β and -γ chains could be rescued in cells treated with cholesterol-saturated MβCD (sMβCD) [11]. Store-operated calcium entry (SOCE) is associated with (Lo)-like regions containing cholesterol [12,13]. This could be related to the presence of cholesterol-binding domains in the critical components of SOCE, namely, the ORAI1 subunit of the calcium-release-activated calcium (CRAC) channel and its regulator, stromal interaction molecule 1 (STIM1) [14,15,16].

Aliphatic chain alcohols are potent membrane fluidizers with anesthetic properties [17,18]. Their amphipathic structure allows them to displace cholesterol from their phospholipid complexes and partially project it into the aqueous environment, thereby stimulating interaction with cholesterol oxidase, saponin, or cyclodextrin. This process is known as cholesterol activation [18,19]. Alcohols can interact with proteins and interfere with their functions without lipid context, as shown in experiments with inhibition of firefly luciferase activity in vitro [20,21]. Lipid-centric and protein-centric hypotheses describe the respective abilities of alcohols to interfere with the functions of lipids and proteins [22]. The biological properties of alcohols depend on the length of the aliphatic chain and the position of the hydroxyl group [23]. The potency correlates with increasing chain length, reflecting their increasing hydrophobicity. However, depending on the system being studied, the sudden cutoff effect could appear, and the opposite response could be observed with a further increase in acyl chain length, as shown in the study comparing the bilayer-modifying potency of 27 aliphatic alcohols using a gramicidin-based fluorescence study [23]. Aliphatic alcohols occur in plants and as pheromones in insects [24,25,26]; they are widely used as cosmetics, detergents, solvents, fuels, pharmaceuticals, and as precursors for polymers and waxes [24]. Although it is known that acyl chain alcohols alter certain properties of the plasma membrane and interfere with the signal transduction in macrophages [25,26] and T cells [27], their effect on mast cells has not been determined. Mast cells are critical players in allergies, innate immunity, and inflammatory reactions. They reside as sentinel cells in the skin and mucosa, where they are capable of releasing many preformed or de novo-produced pro-inflammatory mediators in response to various stimuli [28,29]. Therefore, understanding their sensitivity to alkanols may have practical implications.

We chose 1-heptanol for our study for two reasons. First, we recently described the effect of pentacyclic triterpenoid ursolic acid, a potential anti-inflammatory therapeutic agent, on BMMC signaling. We found that ursolic acid froze the lateral diffusion of cholesterol and FcεRI in resting cells. These events were followed by decreased degranulation and calcium mobilization along with enhanced production of tumor necrosis factor (TNF)-α in antigen-activated BMMCs. Phosphorylation of the FcεRI-β chain, SYK at Tyr^519/520^, and PLCγ1 at Tyr^783^ was increased in these cells [30]. This complex mast cell response in the presence of ursolic acid led us to study mast cells treated with a membrane-fluidizing agent. Recent studies have suggested that 1-heptanol could interfere with cell functions opposite to ursolic acid. This alkanol was used to evaluate the preference of proteins or lipids for Lo-like or Ld-like domains [31]. The authors of this study showed that green fluorescent protein (GFP) linked to the GPI anchor expressed in SH-SY5Y cells, increased lateral diffusion in the presence of 1-heptanol, and formed segregated clusters, which was in contrast to the control cells. Furthermore, truncated LAT1 with a wild-type transmembrane domain and short cytosolic sequence formed clusters in the presence of 1-heptanol; however, the mutated construct in all three transmembrane isoleucines (removing palmitoylation) was randomly distributed even in the presence of 1-heptanol [31]. Second, prior studies have shown that among the aliphatic chain alcohols (C_1_–C_12_), 1-heptanol is the most potent inhibitor of the p65 subunit of nuclear factor (NF) κ-light-chain-enhancer of activated B cells (NF-κB) translocation into the nuclei of peripheral T cells after T cell receptor (TCR) crosslinking. Translocation of NF of activated T cells (NFAT) in the nuclei of primary T cells activated with phorbol 12-myristate 13-acetate/ionomycin was inhibited by 1-heptanol as well. These events were accompanied by dose-dependent inhibition of IFN-γ production in TCR-stimulated primary T cells treated with 1-heptanol [27].

Because mast cell cytokine production is NF-κB and NFAT-dependent [32,33,34,35], we examined the role of 1-heptanol in FcεRI signaling. We studied tyrosine phosphorylation of the FcεRI-β chain and several other signal-transduction molecules, calcium response, degranulation, HSP70 expression, and production of IL-6, TNF-α, and ROS. In addition, we examined 1-heptanol-induced changes in membrane properties, such as thermal stability, lateral mobility of FcεRI, and ORAI1-mCherry and the dynamics of STIM1-ORAI1 interactions as measured by flow cytometry-based Försters resonance energy transfer (flow-FRET). Our data suggest that 1-heptanol-mediated membrane fluidization does not interfere with the earliest biochemical steps of FcεRI signaling, such as phosphorylation of the FcεRI-β chain and components of the SYK/LAT/PLCγ1 signaling pathway, instead inhibiting the FcεRI internalization and mast cell effector functions, including degranulation and cytokine production.

## 2. Materials and Methods

### 2.1. Antibodies and Reagents

The following antibodies and their conjugates were used: monoclonal antibody (mAb) rabbit anti-human p-SYK (Tyr^525^/Tyr^526^; #2710), anti-p-stress-activated protein kinase/c-Jun N-terminal kinase (p-SAPK/JNK; Thr^183^/Tyr^185^; #9255), anti-p-p38 (Thr^180^/Tyr^182^; #4511), and anti-p-IκB-α (Ser^32^/Ser^36^; #9246) were purchased from Cell Signaling Technology (Danvers, MA, USA). Anti-GRB2 (sc-255), anti-actin (sc-8432), anti-extracellular signal-regulated kinase (ERK, sc-93), anti-p-ERK (Tyr^204^; sc-7976), anti-phospholipase C (PLC)γ1 (sc-81), anti-p-PLCγ1 (Tyr^783^; sc-12943), anti-p38 (sc-535), anti-JNK1 (sc-571), anti-IκB-α (sc-371), anti-HSP70 (sc-32239), anti-HSP90 α/β (sc-13119), horseradish peroxidase (HRP)-conjugated goat anti-mouse IgG, goat anti-rabbit IgG, and donkey anti-goat IgG were obtained from Santa Cruz Biotechnology (Dallas, TX, USA). HRP-conjugated anti-phosphotyrosine-specific mouse mAb PY-20 (#610012), and phycoerythrin (PE)-conjugated rat mAb recognizing TNF-α (#554419) were purchased from BD Biosciences (San Jose, CA, USA). Antibody specific for p-LAT1 (Tyr^191^; #07-278; equivalent to Tyr^195^ of mouse LAT1) was obtained from Sigma-Aldrich, St. Louis, MO, USA (Merck, Rahway, NJ, USA). The following antibodies were produced in our laboratory from hybridoma cell lines: IgE mAb specific for 2,4,6-trinitrophenol (TNP), clone IGEL b4 1 [36], SYK-specific mAb [37], anti-FcεRI β chain, clone JRK mAb [38], and anti-LAT1 mAb [39]. Anti-IgE polyclonal antibody was prepared by immunization of rabbits with IGEL b4.1 mAb. Dinitrophenyl (DNP)-specific IgE was a kind gift from Bridget Wilson [40]. TNP-bovine serum albumin (BSA) conjugate (15 to 25 mol TNP/mol BSA) was produced as described previously [41], and DNP-BSA was obtained from Molecular Probes (Thermo Fischer Scientific, Waltham, MA, USA). IgE-fluorescein-5-isothiocyanate (FITC) conjugate was prepared as previously described [42]. Brefeldin A (sc-200861), VER 155008 (sc-358808), and heat shock protein (HSP) I inhibitor (sc-221709) were obtained from Santa Cruz Biotechnology. Recombinant murine stem cell factor (SCF) and interleukin (IL)-3 were obtained from PeproTech EC (Thermo Fischer Scientific, Waltham, MA, USA). Goat anti-mouse IgG-Alexa Fluor^TM^ 514 (H + L; A-31555), thapsigargin (T7459), Fura-2 acetoxymethyl ester (Fura-2-AM; #F1221), and 2′,7′-dichlorodihydrofluorescein diacetate (H_2_DCFDA; C6827) were from Thermo Fisher Scientific, Waltham, MA, USA. Real-time (RT)-polymerase chain reaction (PCR) reagents were obtained from Top-Bio (Vestec, Czech Republic). Annexin V-allophycocyanin (APC; EXB0028) and propidium iodide (PI; EXB0018) were purchased from EXBIO Praha (Vestec, Czech Republic). 1-Heptanol (H2805), saponin (47036), probenecid (P8761), dimethylsulfoxide (DMSO; D8418), Hoechst 33258, 4-methylumbelliferyl-β-D-glucuronide hydrate (M9130), and all other reagents were from Merck.

### 2.2. Mice and Cells

Mice were bred and maintained in a pathogen-free facility of the Institute of Molecular Genetics, used in compliance with the Institute guidelines and as approved by the Institute Commission for Animal Welfare and Protection (Approval No. 72744021). Precursors of BMMC were isolated from the femurs and tibias of 6-to-8-week-old mice of the C57Bl/6J genotype. BMMCs were cultured for 8 to 12 weeks in a complete growth medium containing 10% (*v*/*v*) fetal calf serum (FCS), recombinant mouse IL-3 (15 ng/mL), and recombinant mouse SCF (15 ng/mL). RBL cells, clone 2H3 [43], were grown as an adherent culture [44]. The human embryonic kidney 293FT (HEK293) cell line was provided by the laboratory of Dr. J. Rivera (National Institutes of Health, Bethesda, MD, USA) and cultivated as an adherent culture in Dulbecco’s modified Eagle‘s medium (D6429; Sigma-Aldrich) supplemented with antibiotics (100 U/mL penicillin, 100 μg/mL streptomycin), 10% FCS (Biosera, Cholet, France; catalog no. FB-1090/500), and 2.5 mM L-glutamine. Human c-KIT-positive mast cell line ROSA was a kind gift of Dr. Michel Arock (Laboratoire d’Hématologie Biologique, Hôpital Pitié-Salpêtrière, Paris, France). ROSA cells were grown as a floating culture as described in [45].

### 2.3. Cell Activation

BMMCs were sensitized with TNP-BSA-specific mouse IgE (clone IGEL b4.1) at final concentration 1 μg/mL in SCF- and IL-3-free culture medium for 16 h unless stated otherwise. Cells were washed and activated in buffered saline solution (BSS; 135 mM NaCl, 5 mM KCl, 1.8 mM CaCl_2_, 5.6 mM glucose, 20 mM HEPES buffer, pH 7.4) supplemented with 0.1% essentially fatty acid-free and globulin-free BSA (Sigma; A7030; BSS-BSA). FcεRI-bound IgE was crosslinked with antigen (TNP-BSA conjugate). Alternatively, activation of BMMCs incubated overnight without stem cell factor and IL-3 or HEK293 cells was induced with thapsigargin (1 µM in BSS-BSA), an inhibitor of the sarco/endoplasmic reticulum (ER) Ca^2+^-ATPase (SERCA) pump [46]. In the FcεRI internalization study, DNP-specific mouse IgE was used to sensitize BMMCs, and the bound IgE was crosslinked with DNP-BSA.

### 2.4. Annexin V-APC and Hoechst 33258 Staining

The annexin V binding assay was used to quantify phosphatidylserine surface expression in BMMCs, RBL-2H3 cells, and ROSA cells treated with various concentrations of 1-heptanol. The assay was performed in a 96-well plate. Cells (BMMCs and ROSA cells, 0.25 × 10^6^; RBL-2H3 cells, 0.125 × 10^6^) in 100 μL BSS-BSA were exposed to various concentrations of 1-heptanol for 15 min at 37 °C, centrifuged at 4 °C, and washed with ice-cold BSS-BSA. Cell pellets were suspended in 100 μL of ice-cold modified BSS-BSA with 2.5 mM CaCl_2_, supplemented with 1 μL annexin V-APC, and incubated for 15 min on ice in the dark; 10 μL of Hoechst 33258 was added 5 min before flow cytometry analysis. Data were collected using FACSymphony and BD FACSDiva v9.0 software (BD Biosciences, Franklin Lakes, NJ, USA). The intercalated Hoechst 33258 was excited at 405 nm (emission 450/50 nm) and the fluorescence of the bound annexin V-APC was excited at 637 nm (emission 670/30 nm). Data were analyzed using FlowJo V10 (BD Biosciences).

### 2.5. Thermal Membrane Instability

BMMCs, RBL-2H3 cells, or ROSA cells in BSS-BSA were plated in 96-well white plates for RT-PCR (Roche Diagnostics, Basel, Switzerland; 0.1 × 10^6^ cells/well) in the presence or absence of the indicated concentration of 1-heptanol. For each cell type, 0.05% saponin was used as a positive control. PI at 10 µg/mL (final concentration) was added to all cells, and plates were sealed with LightCycler 480 sealing foil (Roche Diagnostics). The cells were incubated for 15 min at 37 °C before a continuous temperature increase. The rate of temperature increase was 0.01 °C per second. PI fluorescence was measured with a LightCycler 480 instrument (Roche Diagnostics). Alternatively, HSP70 inhibitor VER 155008 and HSP I inhibitor dissolved in DMSO were diluted in the culture medium to final concentrations of 40 μM and 25 μM, respectively, and incubated with the cells overnight. The corresponding concentration of DMSO in the culture medium was used as a control. Data were analyzed using two-way ANOVA. Values on the y axis represent the increased influx of PI into the cells due to the membrane disruption caused by increasing temperature over time and its intercalation into DNA.

### 2.6. Fluorescence Recovery after Photobleaching (FRAP) Measurement

FRAP analysis of resting RBL-2H3 cells labeled with IgE-FITC was performed using a Leica Stellaris 8 Falcon microscopic system equipped with an oil immersion objective (HC PL APO CS2 63×/1.40 oil), with an environmental chamber controlling CO_2_ concentration of 5% and temperature of 37 °C. The cells were grown in glass-bottomed wells of a 96-well black plate (Cellvis, Mountain View, CA, USA; P96-1-N); 10 min before measurement, the cells were treated or not (control) with 2.5 mM 1-heptanol. Four iterations (images) were acquired before photobleaching initiation (time/iteration = 0.276 s). Photobleaching was achieved using 3 × 0.276 s laser pulse at 488 nm with 100% laser power level. Fluorescence intensity was monitored in a circular area 3 μm in diameter (post-bleach 1 = 10 iterations, time/iteration = 0.276 s; post-bleach 2 = 10 iterations, time/iteration = 0.5 s; post-bleach 3 = 30 iterations, time/iteration = 2.5 s). In the experiments using HEK293 cells overexpressing human ORAI1 (BC015369; Open Biosystems; cloned into pmCherry-N1 obtained from Clontech Takara Bio, Kusatsu, Shiga, Japan; ORAI1-mCherry), photobleaching was achieved by 5 × 0.069 s laser pulse at 590 nm at 100% laser power level. Fluorescence intensity was monitored in a circular area 3 μm in diameter (post-bleach 1 = 4 iterations, time/iteration = 0.069 s; post-bleach 2 = 15 iterations, time/iteration = 1 s; post-bleach 3 = 30 iterations, time/iteration = 2 s). Confocal images were obtained using an oil immersion objective (HC PL APO CS2 40 × 1.30 oil) and analyzed as described in [30].

### 2.7. Immunoprecipitation and Immunoblotting

The cells were sensitized with TNP-specific IgE. After washing off unbound IgE, the cells were exposed to 2.5 mM 1-heptanol for 15 min at 37 °C and then activated with antigen (TNP-BSA, 0.5 µg/mL) for selected time intervals. After triggering, the cells were solubilized in an ice-cold immunoprecipitation buffer containing 0.2% Triton X-100 and the selected proteins were immunoprecipitated and analyzed as described in [30]. Tyrosine phosphorylated proteins were analyzed by immunoblotting with PY-20-HRP conjugate or phosphoprotein-specific antibodies, followed by HRP-conjugated secondary antibodies. Luminescent Image Analyzer LAS-3000 (Fujifilm, Tokyo, Japan) and AIDA image analyzer software, v.5.0 (Elysia Raytest, Angleur, Belgium) were used to quantify the HRP signal. Levels of phosphorylated proteins were normalized to loading controls run in parallel gels [47].

### 2.8. Calcium Mobilization and Degranulation

IgE-sensitized cells were loaded for 30 min at 37 °C with 1 µM Fura-2-AM and 2.5 mM probenecid used to prevent dye leakage. After this step, the cells were washed with BSS-BSA and 2.5 mM probenecid and resuspended in BSS-BSA/probenecid and 2.5 mM 1-heptanol or vehicle (BSS-BSA). After incubation for 15 min at 37 °C, the cells were washed in BSS-BSA/probenecid; immediately before measurement, the cells were centrifuged at 200× *g* for 2 min and resuspended in BSS-BSA with 2.5 mM 1-heptanol or vehicle. Aliquots of 50 µL containing 0.2 × 10^6^ cells were added to the wells of a white 96-well plate. BMMCs were activated after 70 s with 50 μL of BSS-BSA supplemented with TNP-BSA (0.5 µg/mL) or thapsigargin (1 µM). In some experiments, the cells were activated with antigen in Ca^2+^-free BSS-BSA and Ca^2+^ was added later after initial triggering, as indicated in the Section 3. HEK293 cells were activated in BSS-BSA with thapsigargin as described above. Changes in intracellular Ca^2+^ concentration ([Ca^2+^]i) were determined by spectrofluorometry using an Infinite 200 M plate reader (TECAN, Männedorf, Switzerland) with excitation wavelengths at 340 and 380 nm and with constant emission at 510 nm. Data are presented as the ratio em510_exc340_/em510_exc380_. The extent of degranulation was evaluated by determining β-glucuronidase release into supernatant as previously described [47].

### 2.9. Flow Cytometry-Based FRET

Human cDNA for STIM1 (BC021300) and ORAI1 (BC015369) were purchased from Open Biosystems. The following primers were used to amplify STIM1 and ORAI1: Stim1_forward: 5′AAAGAATTCGCTAGCACCATGGATGTATGCGTCCGTC3′, Stim1_reverse: 5′TTTGGATCCCGCTTCTTAAGAGGCTTCTTAAAG3′; Orai1_forward: 5′AAAGAATTCGCTAGCACCATGCATCCGGAGCCCGCCCCG3′, Orai1_reverse: 5′TTTGGATCCCGGGCATAGTGGCTGCCGGGCG3′. PCR fragments with introduced restriction site EcoRI in forward primers and BamHI in reverse primers (underlined) were cloned into the MCS regions of enhanced cyan fluorescent protein (ECFP)- or enhanced yellow fluorescent protein (EYFP)-N1 vectors (Clontech) using corresponding restriction enzymes. EYFP-ECFP ligand was similarly generated by cloning ECFP into EYFP-C1 (Clontech). Polyethylenimine (25 kDa; linear form; Polysciences)-mediated transfection of HEK293 cells with 2.5 µg and 1.5 µg of plasmids containing ECFP and EYFP proteins, respectively, mixed in Opti-MEM medium (Thermo Fisher Scientific) was performed 24 h before the experiments. The flow-FRET method was used as previously described in [48]. A detailed description of the gating strategy is provided in the Section 3.

### 2.10. Determination of the mRNA Expression Levels

IgE-sensitized BMMCs were pretreated with 2.5 mM 1-heptanol for 15 min at 37 °C or left alone and then activated or not with antigen (TNP-BSA; 0.5 µg/mL). One hour later, mRNA was extracted using RNA Blue reagent (Top-Bio). cDNA was synthesized using M-MLV reverse transcriptase and random hexamer primers following the manufacturer’s instructions (Invitrogen, Waltham, MA, USA). RT-PCR amplifications of cDNAs were performed in 10 μL reaction volumes of a quantitative PCR mix containing 1 M 1,2-propanediol, 0.2 M trehalose, and SYBR Green 1 [49], commercially available from Top-Bio, then analyzed in a qTower^3^G cycler (Analytik Jena). The following cycling conditions were used: 3 min at 95 °C, followed by 45 cycles of 10 s at 95 °C, 10 s at 60 °C, and 12 s at 72 °C. Cytokine mRNA expression levels are reported as the fold-change relative to the cytokine mRNA expression level of untreated and non-activated cells. HSP70 and HSP90 mRNA levels were detected in non-activated cells. Target gene data were normalized to the geometric mean of the reference genes hypoxanthine-guanine phosphoribosyltransferase (HPRT) and ubiquitin (UB). Primer sequences are as follows:

TNF-α: 5′CCCTCACACTCAGATCATCTTCT3′, 5′GCTACGACGTGGGCTACAG3′; IL-6: 5′GAGGATACCACTCCCAACAGACC3′, 5′AAGTGCATCATCGTTGTTCATACA3′; HSP70: 5′CAGCGAGGCTGACAAGAAGAA3′, 5′GGAGATGACCTCCTGGCACT3′; HSP90: 5′AAACAAGGAGATTTTCCTCCGC3′, 5′CCGTCAGGCTCTCATATCGAAT3′; HPRT: 5′CTGGTGAAAAGGACCTCTCGAA3′, 5′CTGAAGTACTCATTATAGTCAAGGGCAT3′; UB: 5′ATGTGAAGGCCAAGATCCAG3′, 5′ TAATAGCCACCCCTCAGACG3′.

### 2.11. Bead-Based Immunoassay for Assessment of Cytokine Secretion

IgE-sensitized BMMCs (1 × 10^6^ cells per well) were pretreated with or without 2.5 mM 1-heptanol for 15 min, followed by activation with antigen (TNP-BSA; 0.5 µg/mL) diluted in BSS-BSA (control) or in BSS-BSA with 2.5 mM 1-heptanol for 4 h. Then, the cells were centrifuged and the supernatants (25 µL) were used for cytokine quantification using a bead-based immunoassay (LEGENDplex™ multi-analyte flow assay; mouse B effector panel 8-plex; cat No. 740820; BioLegend, San Diego, CA, USA) according to the manufacturer’s instructions. Data were acquired using a BD Symphony flow cytometer with 488 and 637 nm lasers. Data were analyzed using LEGENDplex™ data analysis software v. 8.0.

### 2.12. ROS Measurements

ROS were assessed using cell-permeant H2DCFDA as a reporter, as described previously [50], with modifications. Briefly, IgE-sensitized cells (0.25 × 10^6^ cells/well) were cultured in 100 μL of medium supplemented with H2DCFDA (5 μg/mL), 2.5 mM probenecid in the absence or presence of 2.5 mM 1-heptanol. After 15 min the cells were centrifuged and resuspended in 50 μL BSS-BSA with or without 1-heptanol. Activation was induced by adding 50 μL of TNP-BSA (final concentration 0.5 µg/mL) in the absence or presence of 2.5 mM 1-heptanol. Alternatively, in the experiments with arachidonic acid, cells were stained with H2DCFDA as above, washed, and incubated for 15 min in BSS-BSA supplemented or not with 2.5 mM 1-heptanol and 80 µM arachidonic acid. After 10 min, the cells were activated with antigen (TNP-BSA, 0.5 µg/mL); changes in fluorescence intensity were monitored using a FACSymphony flow cytometer and BD FACSDiva software in the appropriate FITC channel (BD Biosciences; Ex/Em 488/505–535 nm). Data were analyzed using FlowJo V10 (BD Biosciences).

### 2.13. FcεRI Internalization

The relative amount of surface FcɛRI on BMMCs during their activation by DNP-BSA was determined by flow cytometry as previously described [51]. Briefly, BMMCs were sensitized by exposure to DNP-specific IgE (1 µg/mL). After overnight incubation, the cells were washed and treated with 2.5 mM 1-heptanol or DMSO at 37 °C. After 15 min, the cells were activated with DNP-BSA (0.5 µg/mL) for the indicated time intervals in the absence (Control) or presence of 2.5 mM 1-heptanol during the entire time-course. Activation was terminated by a fixation step with 2% paraformaldehyde for 10 min at room temperature. After washing, FcɛRI-bound DNP-specific IgE was labeled with Alexa Fluor 514-conjugated goat anti-mouse IgG (cross-reacting with IgE). Changes in fluorescence intensity were monitored using FACSymphony flow cytometer and BD FACSDiva software. Data analysis was performed using FlowJo V10 (BD Biosciences). The fluorescence intensity of non-activated and 1-heptanol-untreated cells was set to 100%; relative FcɛRI expression was calculated at the indicated activation times.

### 2.14. Quantification of Arachidonic Acid and Leukotriene (LT)C4

BMMCs (1 × 10^6^) were sensitized with TNP-specific IgE (1 μg/mL) in SCF- and IL-3-free culture medium for 16 h. The cells were then washed in BSS-BSA and activated with antigen (TNP-BSA; 0.5 μg/mL) in the absence or presence of 2.5 mM 1-heptanol for 30 min. Cell supernatants were collected and immediately frozen in liquid nitrogen. UPLC MS/MS analysis was performed as described elsewhere [35].

### 2.15. Measurement of Cellular TNF-α

IgE-sensitized BMMCs were washed, added to the wells of a 96-well plate (0.25 × 10^6^ cells per well), and incubated with brefeldin A (5 μg/mL). After 2 h, the cells were treated with 2.5 mM 1-heptanol or not (control) for 15 min, followed by activation with antigen (TNP-BSA; 0.5 µg/mL) for 1.5 h in the presence of brefeldin A (5 μg/mL) alone or brefeldin A (5 μg/mL) and 2.5 mM 1-heptanol, centrifuged at 200× *g* for 3 min at 22 °C, and fixed with 4% paraformaldehyde in PBS for 15 min at 22 °C. After washing in BSS-BSA, cells were permeabilized with 0.1% saponin in BSS-BSA for 15 min at 22 °C, centrifuged as above, and resuspended in BSS-BSA supplemented with anti-TNF-α-PE conjugate for 1 h. After washing in BSS-BSA, fluorescence intensity was monitored using a FACSymphony flow cytometer and BD FACSDiva software in the appropriate PE channel (BD Biosciences; Ex/Em 561/571–601 nm). Data analysis was performed using FlowJo V10 (BD Biosciences).

### 2.16. Statistical Analysis

Statistical analysis was carried out using Student’s unpaired two-tailed *t*-test to compare two groups. One-way or two-way ANOVA with Tukey’s or Dunnett’s post hoc comparison was used to compare more than three experimental groups. Within-group multiple comparisons were performed. Calculated *p* values of less than 0.05 were considered significant, with significance levels as follows: * *p* < 0.05; ** *p* < 0.01; *** *p* < 0.001; and **** *p* < 0.0001. For each analysis, the data met the assumptions of the statistical test. Data are expressed as mean ± SEM. All statistical analyses were performed using GraphPad Prism v. 7.3. (La Jolla, CA, USA).

## 3. Results

### 3.1. Short-Term Exposure to 1-Heptanol Alters the Properties of the Mast Cell Plasma Membrane and the Mobility of FcεRI

**Figure 1:**
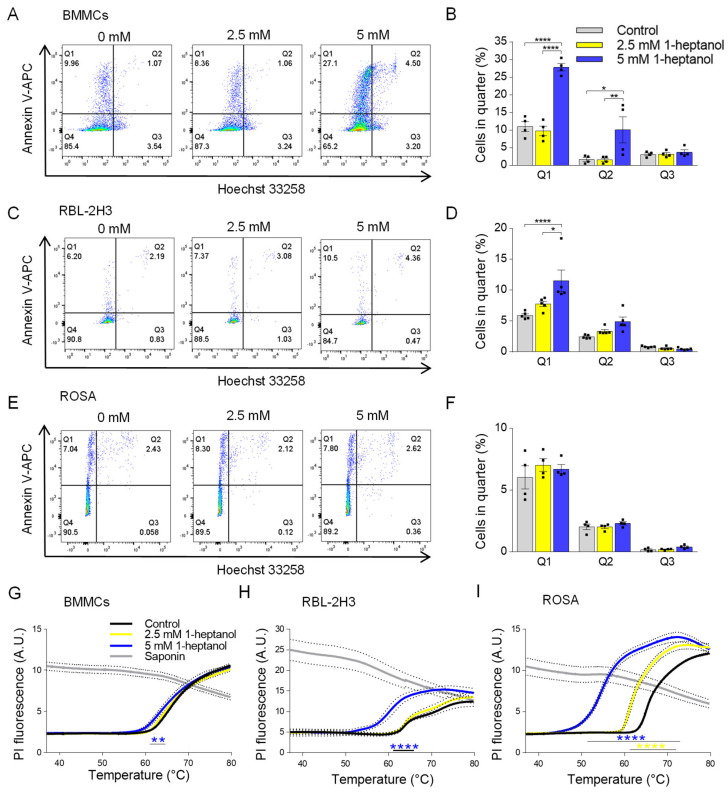
Dose-dependent effects of 1-heptanol on annexin V-APC/Hoechst 33258 staining and membrane thermal stability of various mast cell types. (**A**–**F**) Representative flow cytometry profiles of mouse BMMCs (**A**), RBL-2H3 cells (**C**), and human ROSA cells (**E**) exposed for 15 min to various concentrations of 1-heptanol and stained with annexin V-APC and Hoechst 33258. Quantitative analysis of flow cytometry data as shown in (**A**) ((**B**), *n* = 4), (**C**) ((**D**), *n* = 4), and (**E**) ((**F**), *n* = 4). (**G**–**I**) Membrane thermal stability analysis. BMMCs ((**G**), *n* = 4), RBL-2H3 cells ((**H**), *n* = 3), and human ROSA cells ((**I**), *n* = 4) were exposed to the indicated concentrations of 1-heptanol and PI. The cells were plated in 96-well plates for RT-PCR and incubated for 15 min at 37 °C, followed by PI fluorescence measurement at a slowly increasing temperature in the RT-PCR instrument. Saponin-treated cells with disrupted membranes served as positive controls in (**G**–**I**); these data were not included in the statistical evaluation of intergroup differences using 2-way ANOVA. Values indicate means ± SEM calculated from *n*, which show numbers of biological replicates; * *p* < 0.05, ** *p* < 0.01, **** *p* < 0.0001.

To examine the effect of 1-heptanol on various mast cell types, we exposed murine BMMCs, RBL-2H3 cells, and human ROSA mast cells to 1-heptanol at up to 5 mM concentrations in initial experiments. First, we used flow cytometry to measure markers of apoptosis and cell death, along with phosphatidylserine externalization, using annexin V-APC and Hoechst 33258 DNA staining, respectively (Figure 1A–F). The examined cell types exhibited different levels of basal staining. We found that all examined cell types were well able to tolerate a concentration of 2.5 mM 1-heptanol, as annexin V-APC-dependent phosphatidylserine staining (quarter 1; Q1), Hoechst 33258 intracellular presence (quarter 3; Q3), and double staining (quarter 2; Q2) were comparable with the staining of cells incubated in the absence of 1-heptanol. In contrast to the ROSA cells, BMMCs and RBL-2H3 cells exposed to 5 mM 1-heptanol exhibited an enhanced percentage of cells in Q1. The fraction of BMMCs affected by 5 mM 1-heptanol was enhanced in Q2 as well (Figure 1A–F). It should be noted that in mast cells and basophils the externalization of phosphatidylserine is associated not only with apoptosis but with activation-mediated rearrangement of lipids in the outer leaflet of the plasma membrane [52,53].

Next, we analyzed the dose-dependent effects of 1-heptanol on plasma membrane physical properties. To this end, we developed a new method based on measuring the thermal instability of the plasma membrane (Figure 1G–I). In this assay, the cells are plated in a 96-well plate for RT-PCR in the presence of PI, which is impermeable to live cells. The temperature increase (0.01 °C/s) over time causes the cells to burst at some point, allowing PI to penetrate the cell nuclei and intercalate into DNA. The RT-PCR instrument is used to detect the increase in fluorescence. We found that BMMCs treated with 5 mM 1-heptanol were less stable to increasing temperature than cells treated with 2.5 mM 1-heptanol or control cells (Figure 1G). RBL-2H3 cells showed higher sensitivity to 5 mM 1-heptanol than BMMCs (Figure 1H). ROSA cells showed even higher sensitivity to 5 mM 1-heptanol (Figure 1I). Interestingly, treatment of ROSA cells with 2.5 mM 1-heptanol significantly enhanced the temperature sensitivity (Figure 1I). As positive controls exhibiting maximum staining in this assay, we used saponin, a cholesterol-dependent cytolysin making pores in the plasma membranes [54]. The observed decrease in the fluorescence of PI in saponin-treated cells at higher temperatures is presumably caused by the photobleaching of DNA-intercalated PI (Figure 1G–I). Based on the results of the initial experiments, we used 1-heptanol at a final concentration of 2.5 mM in all subsequent experiments.

**Figure 2:**
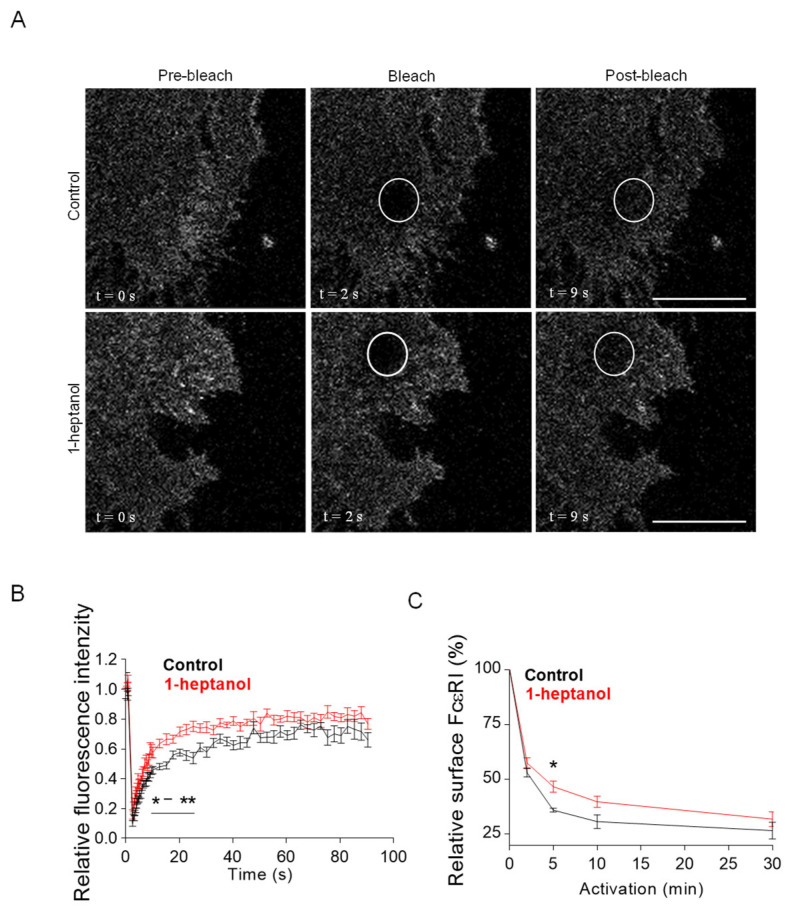
1-Heptanol enhances the lateral mobility of FcεRI, as determined by FRAP, but retards internalization. (**A**) IgE-FITC-sensitized RBL-2H3 cells were exposed for 10 min to the vehicle (BSS-BSA; Control) or 2.5 mM 1-heptanol. Representative fluorescence image sequences are from cells at the indicated time points before photobleaching (Pre-bleach), at photobleaching (Bleach; the circled areas) or after photobleaching (Post-bleach; the circled areas). Bars indicate 10 µm. (**B**) Quantification of the recovery into the photobleached areas as shown in A. The relative fluorescence intensity was calculated at each time point by dividing the fluorescence in the bleached region by the fluorescence in the corresponding unbleached region. The background was subtracted from all signals. Values in (**B**) indicate means ± SEM of two independent experiments for the control cells (*n =* 16) and 1-heptanol-treated cells (*n* = 15). (**C**) FcεRI internalization in antigen-activated BMMCs in the absence (control, *n* = 5) or presence of 2.5 mM 1-heptanol (*n* = 5). Statistical significance of intergroup differences is indicated in (**B**,**C**); * *p* < 0.05, ** *p* < 0.01.

The amphipathic properties of 1-heptanol result in its efficient intercalation into the cell plasma membrane and activation of membrane cholesterol [18]. This process is accompanied by exposure of cholesterol to its oxidases [18,19] and hyperfluidization of cell membranes associated with domain segregation [31]. Because cholesterol in the membrane is crucial for proper FcεRI signaling [11,50,54], we investigated the mobility of FcεRI in the plasma membrane of 1-heptanol-treated cells. For these experiments, we used RBL-2H3 cells labeled with IgE-FITC and measured the dynamics of FcεRI diffusion with FRAP (Figure 2A). Data summarized in Figure 2B show that FcεRI mobility was significantly enhanced in cells exposed to 2.5 mM 1-heptanol at 9–27 s after photobleaching. Quantification of the acquired FRAP curves yielded an FcεRI diffusion coefficient of 0.16 ± 0.06 µm^2^ s^−1^ in control cells, compared to 0.32 ± 0.1 µm^2^ s^−1^ in 1-heptanol-treated cells; the dissociation (k_off_) rate was 0.19 ± 0.04 s^−1^ in control cells and 0.24 ± 0.03 s^−1^ in cells exposed to 1-heptanol. Consistently, 31.40 ± 3.91% of FcεRI was present in the immobile fraction in control cells compared to 23.50 ± 2.85% in 1-heptanol exposed cells.

FcεRI is rapidly internalized after its aggregation with IgE-multivalent Ag complexes [55,56]. Therefore, we next investigated the internalization of antigen-aggregated FcεRI in BMMCs untreated or treated with 2.5 mM 1-heptanol by quantifying the amount of FcεRI on the plasma membrane after IgE crosslinking with multivalent antigen (Figure 2C).

### 3.2. 1-Heptanol Has No Significant Effect on Enhanced Tyrosine Phosphorylation Observed in Early Stages in Antigen-Activated Cells

**Figure 3:**
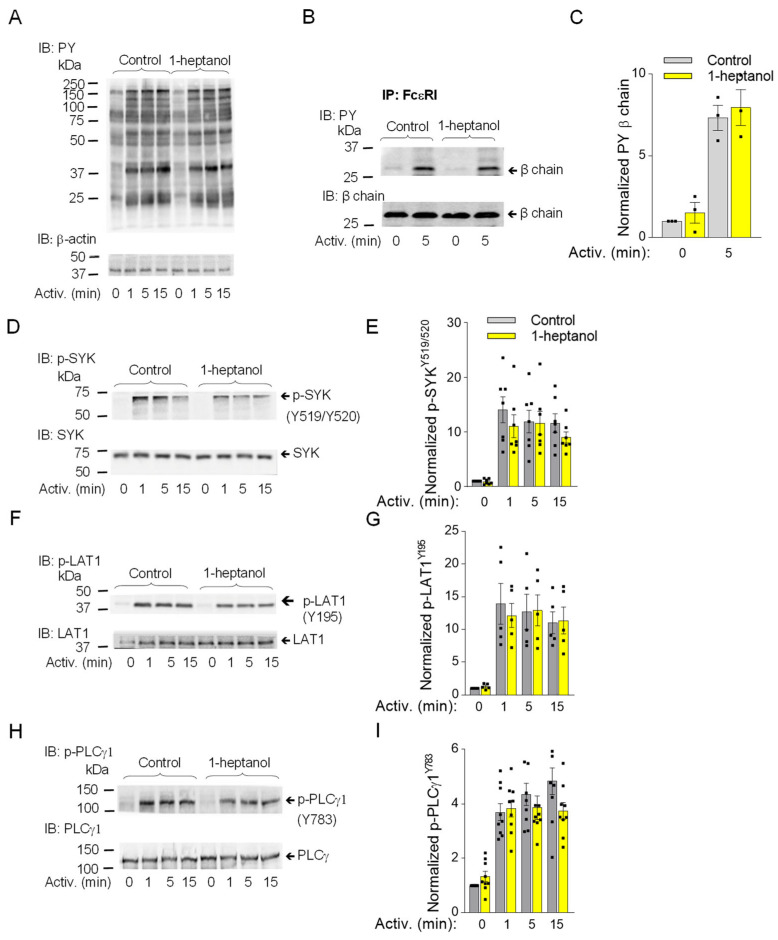
1-Heptanol does not affect tyrosine phosphorylation of global cellular proteins, FcεRI-βsubunit, or SYK/LAT1/PLCγ1 axis in antigen-activated BMMCs. (**A**) IgE-sensitized BMMCs were exposed for 15 min to the vehicle (BSS-BSA; Control) or 2.5 mM 1-heptanol and then activated with antigen (TNP-BSA; 0.5 µg/mL) for the indicated time intervals in the absence or presence of 2.5 mM 1-heptanol. Whole lysates from the cells were size-fractionated by SDS-PAGE and analyzed by immunoblotting (IB) with PY-20-HRP conjugate. For loading control, the blots were developed with an antibody specific for β-actin (*n* = 3). (**B**,**C**) The cells were treated for 15 min with vehicle (Control) or 2.5 mM 1-heptanol and then activated with antigen as in A. The FcεRI complexes were immunoprecipitated (IP) and analyzed by IB with the PY-20-HRP conjugate. The position of the FcεRI-β chain is indicated (*n* = 3). (**D**–**I**) IgE-sensitized BMMCs were treated and activated as in A; the whole-cell lysates were size-fractionated by SDS-PAGE and analyzed by immunoblotting with phosphotyrosine-specific antibodies recognizing SYK^Y519/Y520^ ((**D**,**E**); *n* = 7), LAT1^Y195^ ((**F**,**G**); *n* = 5), and PLCγ1^Y783^ ((**H**,**I**); *n* = 9). Representative immunoblots with the corresponding protein loading controls are shown in B, D, F, and H. The results from quantifying the tyrosine-phosphorylated proteins in activated cells are normalized to signals in non-activated cells and loading control proteins (**C**,**E**,**G**,**I**). Values indicate means ± SEM calculated from the numbers indicated above (n), which denote the numbers of biological replicates. Numbers on the left in (**A**,**B**,**D**,**F**,**H**) indicate the positions of molecular weight markers in kDa.

The observed changes in plasma membrane properties in 1-heptanol-treated cells led us to examine global tyrosine phosphorylation of non-activated or antigen-activated cells. The lysates from BMMCs activated for the indicated time intervals (Figure 3A) in the presence of vehicle (BSS-BSA) or 2.5 mM 1-heptanol were size-fractionated by SDS-PAGE, followed by immunoblotting with PY-20-HRP. No difference between control and 1-heptanol-treated cells was noticed in the tyrosine phosphorylation of total cellular proteins (Figure 3A). The earliest detectable biochemical step in FcεRI signaling is Lyn-mediated phosphorylation of the immunoreceptor tyrosine-based activation motif (ITAM) of the FcεRI-β chain [3]. To determine the effect of 1-heptanol on the FcεRI phosphorylation, we immunoprecipitated the FcεRI-IgE complex from non-activated or antigen-activated BMMCs pretreated with vehicle or 2.5 mM 1-heptanol. Immunocomplexes were size-fractionated by SDS-PAGE and analyzed by immunoblotting with a phosphotyrosine-specific antibody PY-20-HRP conjugate. We found that pretreatment of the cells with 2.5 mM 1-heptanol did not affect tyrosine phosphorylation of FcεRI-β ITAM (Figure 3B,C).

The aggregation of FcεRIs results in recruitment, rapid phosphorylation and activation of SYK, a critical protein tyrosine kinase that transduces extracellular signals to downstream targets [57]. The activation loop of rodent SYK contains Tyr^519^ and Tyr^520^ (equivalent to Tyr^525^ and Tyr^526^ of human SYK), which are not essential for the enzymatic activity of SYK but are required for the propagation of FcεRI-mediated signaling leading to the initiation of cell effector functions [58]. SYK phosphorylates many substrates, including LAT1, an adaptor protein that is a positive regulator of mast cell signaling [59,60]. LAT1 contains several functional tyrosine sites, including Tyr^195^ in rodents (equivalent to Tyr^191^ of human LAT1), a docking site for the SH2 domains of the cytoplasmic adaptor proteins GRB2 and Gads [61]. SYK phosphorylates PLCγ1 at Tyr^783^, a tyrosine that induces lipase activity [62], and PLCγ1 is a binding partner of LAT1 through its SH2 domain [61]. The FcεRI-β/SYK/LAT1/PLCγ1 signaling axis leads to the PLCy1-catalyzed shedding of inositol 1,4,5-trisphosphate (IP3) from the plasma membrane, which precedes the IP3-mediated opening of the ER membrane-embedded IP3 Ca^2+^ channel (IP3R) and causes the release of intracellularly stored Ca^2+^. These events are followed by the opening of CRAC channels on the plasma membranes and a more sustained influx of Ca^2+^ from the extracellular environment [63,64]. To determine the effect of 1-heptanol on tyrosine phosphorylation of the signaling proteins, we examined SDS-PAGE-fractionated whole-cell lysates from non-activated or antigen-activated BMMCs in the presence or absence of 1-heptanol, followed by immunoblotting with phosphoprotein-specific antibodies. Consistent with the global tyrosine phosphorylation profile, we found comparable phosphorylation of the early markers of FcεRI signaling, SYK^Y519/Y520^, LAT1^Y195^, and PLCγ1^Y783^ in BMMCs treated or untreated with 1-heptanol (Figure 3D–I).

**Figure 4:**
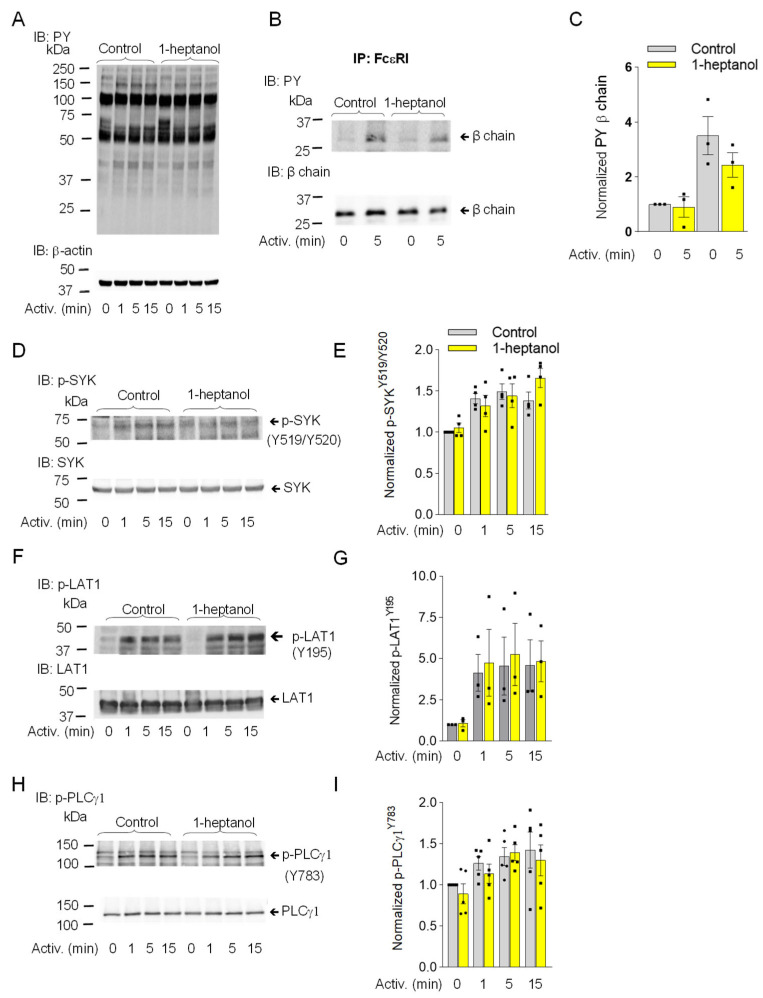
1-Heptanol does not affect tyrosine phosphorylation of global cellular proteins, FcεRI-βsubunit, or the SYK/LAT1/PLCγ1 axis in antigen-activated RBL-2H3 mast cells. (**A**) IgE-sensitized cells were treated for 15 min with vehicle (Control) or 2.5 mM 1-heptanol and then activated with antigen (TNP/BSA; 0.5 µg/mL) for the indicated time intervals in the absence or presence of 2.5 mM 1-heptanol. Whole-cell lysates were size-fractionated by SDS-PAGE and analyzed by immunoblotting (IB) with PY-20-HRP conjugate. For loading control, the blots were developed with an antibody specific for β-actin (*n* = 3). (**B**,**C**) The cells were treated for 15 min with vehicle (Control) or 2.5 mM 1-heptanol and then activated with antigen as in **A** for 5 min. The FcεRI complexes were immunoprecipitated (IP) and analyzed by IB with the PY-20-HRP conjugate. For loading controls, the blots were developed with FcεRI-β chain-specific antibody. The position of the FcεRI-β chain is indicated (*n* = 3). (**C**) Quantitative analysis of the phosphorylated FcεRI-β chains normalized to their amounts. Values indicate means ± SEM (*n* = 3). (**D**–**I**) RBL-2H3 cells were activated for different time intervals as in A, size-fractionated by SDS-PAGE, and analyzed by immunoblotting with phosphotyrosine-specific antibodies recognizing SYK^Y519/Y520^ ((**D**,**E**); *n* = 4), LAT1^Y195^ ((**F**,**G**); *n* = 3), and PLCγ1^Y783^ ((**H**,**I**); *n* = 5). The antibodies specific to the corresponding proteins were used as loading controls. Representative immunoblots with the corresponding protein loading controls are shown in (**D**,**F**,**H**). The results from the quantification of the tyrosine-phosphorylated proteins in activated cells are normalized to signals in non-activated cells and loading control proteins (**E**,**G**,**I**). Values indicate means ± SEM calculated from the numbers above (n), denoting the numbers of biological replicates. Numbers on the left in (**A**,**B**,**D**,**F**,**H**) indicate the positions of molecular weight markers in kDa.

Similarly, in RBL-2H3 mast cells, pretreatment with 2.5 mM 1-heptanol did not significantly affect the tyrosine phosphorylation of total cellular proteins (Figure 4A), FcεRI-β ITAM (Figure 4B,C), SYK^Y519^/^Y520^ (Figure 4D,E), LAT1^Y195^ (Figure 4F,G), or PLCγ1^Y783^ (Figure 4H,I).

### 3.3. 1-Heptanol Specifically Inhibits SAPK/JNK Phosphorylation in BMMCs, but Not Other Members of the Mitogen-Activated Protein Kinases, ERK and p38

**Figure 5:**
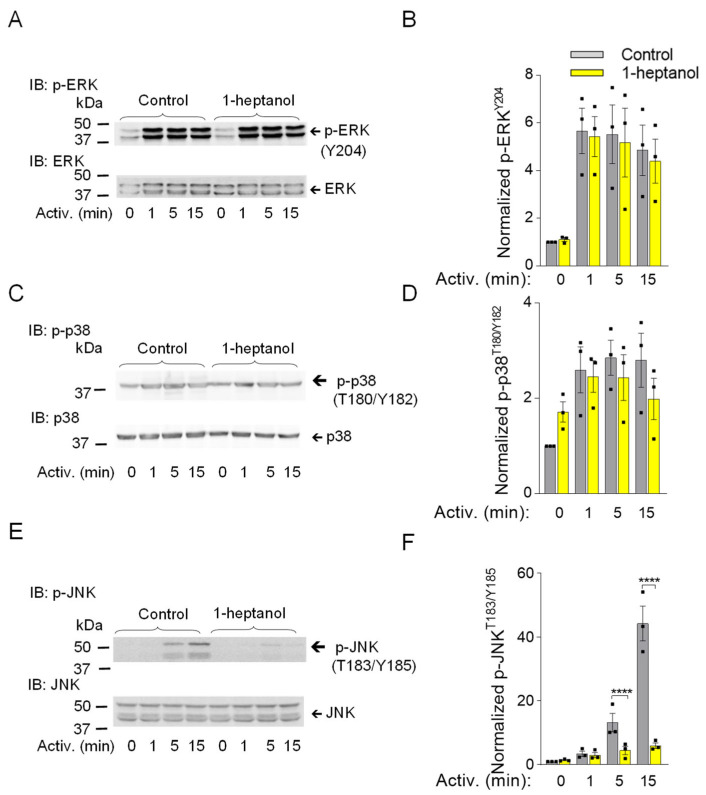
1-Heptanol inhibits phosphorylation of SAPK/JNK (JNK) but not ERK and p38 in antigen-activated BMMCs. (**A**–**F**) IgE-sensitized BMMCs were pre-incubated with vehicle (BSS-BSA; Control) or 2.5 mM 1-heptanol for 15 min and then activated or not with antigen (TNP-BSA; 0.5 µg/mL) for the indicated time intervals. Whole-cell lysates were size fractionated by SDS-PAGE and analyzed by immunoblotting with phosphotyrosine-specific antibodies recognizing ERK^Y204^ ((**A**,**B**); *n* = 3), p38^T180/Y182^ ((**C**,**D**); *n* = 3), and JNK^T183/Y185^ ((**E**,**F**); *n* = 3). Antibodies specific for the corresponding proteins were used as loading controls. Representative immunoblots with the corresponding protein loading controls are shown in (**A**,**C**,**E**). The results of quantification of the tyrosine-phosphorylated proteins in activated cells are normalized to the signals in non-activated cells and loading control proteins (**B**,**D**,**F**). Values are mean ± SEM calculated from the numbers indicated above (n), which indicate the numbers of biological replicates. Statistical significance of intergroup differences is indicated in (**F**); **** *p* < 0.0001. Numbers on the left in (**A**,**C**,**E**) indicate positions of molecular weight markers in kDa.

Mitogen-activated protein kinases (MAPK) ERK, p38, and SAPK/JNK are phosphorylated upon FcεRI aggregation and under stressful conditions such as heat stress or exposure of cells to ultraviolet light, causing them to initiate stress-protective responses [65,66]. Multi-regulatory ERK kinase with phosphorylated Tyr 202 and Tyr 204 in the activation loop is one of the markers of late signaling events [67]. Antigen-activated BMMCs showed no changes in ERK^Y204^ phosphorylation upon exposure to 1-heptanol compared to vehicle-treated cells (Figure 5A,B). Similarly, the dual phosphorylation of p38 at Thr 180 and Tyr 182, which is caused by the response to various stimuli [68], was not altered in antigen-activated BMMCs treated with 1-heptanol compared to untreated cells (Figure 5C,D). In contrast, the presence of 2.5 mM 1-heptanol selectively inhibited the dual activating phosphorylation of SAPK/JNK at Thr 183 and Tyr 185 in antigen-activated BMMCs (Figure 5E,F). Our data indicate that 1-heptanol specifically interferes with SAPK/JNK activation, but not with ERK and P38 MAPK family members, during FcεRI-mediated signal transduction.

### 3.4. 1-Heptanol Inhibits β-Glucuronidase Release and Calcium Mobilization

**Figure 6:**
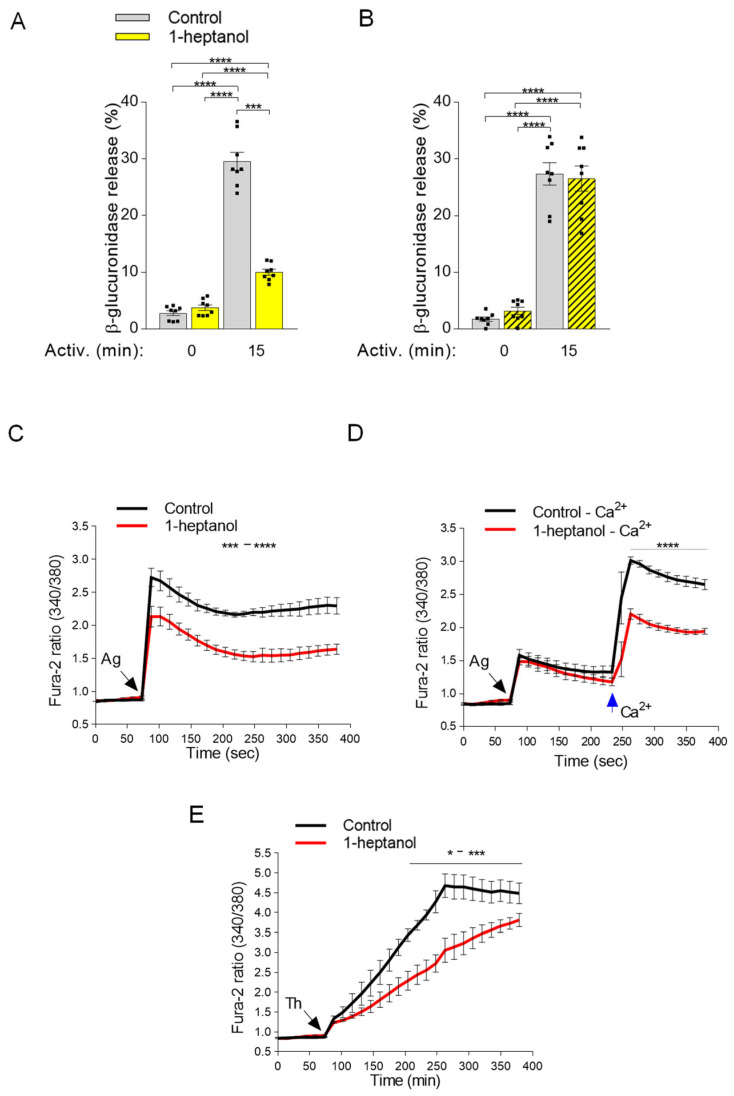
1-Heptanol inhibits mast cell degranulation and calcium mobilization in activated cells. (**A**) IgE-sensitized BMMCs pretreated for 15 min with vehicle (PBS) alone (Control) or 2.5 mM 1-heptanol were non-activated (time 0 min; *n* = 8) or activated for 15 min with antigen (TNP-BSA; 0.5 µg/mL; *n* = 8). The level of β-glucuronidase released into the supernatant was determined using 4-methylumbelliferyl-β-D-glucuronide hydrate as a substrate. (**B**) In the experiment with reversibility of 1-heptanol action, the cells were treated as in A, but 1-heptanol was washed out before antigen activation. (**C**) Intracellular calcium mobilization in IgE-sensitized BMMCs in the presence of extracellular calcium and vehicle (Control) or 1-heptanol. BMMCs were loaded for 15 min in the presence of 1.8 mM calcium with Fura 2-AM alone (Control; black line; *n* = 5) or with 2.5 mM 1-heptanol (red line; *n* = 5). (**D**) Alternatively, the cells were loaded for 15 min with Fura-2-AM in the Ca^2+^-free BSS-BSA in the absence (Control–Ca^2+^; black line; *n* = 5) or presence of 2.5 mM 1-heptanol (red line; *n* = 5). The cells were activated with antigen (Ag, the arrow, TNP-BSA; 0.5 µg/mL) followed by the addition of calcium (final concentration 1.8 mM) at the indicated time interval (blue arrow Ca^2+^). (**E**) Intracellular calcium mobilization was analyzed as in C, except that thapsigargin (Th) at a final concentration of 1 µM was used for triggering (*n* = 4). The extent of calcium mobilization in (**C**,**D**) was monitored by measuring the fluorescence ratios. The bars in (**A**,**B**) indicate statistically significant intergroup differences. The bars above the curves in (**C**,**D**) indicate statistically significant differences between controls and 1-heptanol-treated groups. Values indicate means ± SEM, calculated from *n*, which show numbers of biological replicates; * *p* < 0.05, range of *** *p* < 0.001 **** *p* < 0.0001.

Antigen-activated mast cells secrete preformed bioactive compounds from their granules [29]. This process can be quantified by measuring the levels of released mediators, such as histamine or β-glucuronidase [47,53]. Next, we examined the levels of β-glucuronidase in the supernatants of antigen-activated BMMCs exposed to 2.5 mM 1-heptanol for 15 min prior to antigen addition (Figure 6A). In non-activated cells, the amount of β-glucuronidase was similar in the control and 1-heptanol pretreated cells, indicating no change in spontaneous degranulation. This finding supports the notion that short-term exposure of BMMCs to 2.5 mM 1-heptanol is not toxic or activating for mast cells. In the supernatants of antigen-activated control cells, the amount of β-glucuronidase was increased almost ten-fold (29.6 ± 1.6% SEM). In cells treated with 2.5 mM 1-heptanol and activated by antigen, the amount of β-glucuronidase released from the cells was significantly reduced (10.0 ± 0.5% SEM; Figure 6A). The observed inhibition of degranulation by exposure to 2.5 mM 1-heptanol for 15 min was completely reversible, as inferred from the experiments in which washout of 1-heptanol immediately before antigen addition restored degranulation (Figure 6B). These data are consistent with the literature data showing that 1-heptanol-mediated phase separation of membrane molecules is a reversible process [31].

Calcium is a second messenger essential for intracellular signaling [32]. Therefore, in the next experiment we examined calcium mobilization in antigen-activated BMMCs exposed to 1-heptanol (Figure 6C). We found that antigen-activated BMMCs exposed to 2.5 mM 1-heptanol exhibited a decrease in the calcium response when compared to control antigen-activated cells (Figure 6C). The antigen-activated cells in the medium without extracellular calcium responded with a lower magnitude corresponding to the calcium wave released from intracellular stores. However, no significant difference between 1-heptanol-treated cells and controls was observed (Figure 6D). Addition of extracellular calcium to the control cells resulted in a calcium influx comparable to that seen in cells with a calcium-supplemented medium. In cells treated with 1-heptanol, significant reduction of the calcium response was observed (Figure 6D). Similarly, cells activated with the nonspecific activator thapsigargin [46] exhibited a 1-heptanol-dependent decrease in calcium response (Figure 6E). The combined data suggest that the 1-heptanol-mediated decrease in calcium response is at least in part caused by a defect in the function of calcium channels in the plasma membrane of 1-heptanol-treated cells.

### 3.5. Short-Term Exposure to 1-Heptanol Increases the Lateral Mobility of ORAI1-mCherry and Alters the Dynamics of STIM1-ORAI1 Interaction

Because 1-heptanol had no effect on tyrosine phosphorylation of SYK, LAT1, and PLCγ1 but inhibited Ca^2+^ mobilization, we focused on the mechanical part of the SOCE. CRAC channels comprise ORAI1 dimers in the inactive state and ORAI1 hexamers in cells with empty ER Ca^2+^ stores [63]. Ca^2+^ levels in ER stores are sensed by ER membrane-spanning protein STIM1, which responds to empty Ca^2+^ stores by conformational changes and formation of aggregates [64]. STIM1 clusters are translocated to the vicinity of the plasma membrane, where they interact directly with ORAI1 proteins and trigger the formation of functional Ca^2+^ channels [63,64]. The non-functional SOCE causes the development of severe combined immunodeficiency [69]. In antigen-activated mast cells, deletion or downregulation of STIM1 or ORAI1 inhibits calcium mobilization and Ca^2+^-dependent effector functions, including LTC4 synthesis and degranulation, phosphorylation of IκB-α, translocation of nuclear factor of activated T cells (NFAT) and p65 into the nuclei, cytokine secretion, and reorganization of microtubules [32,70,71].

**Figure 7:**
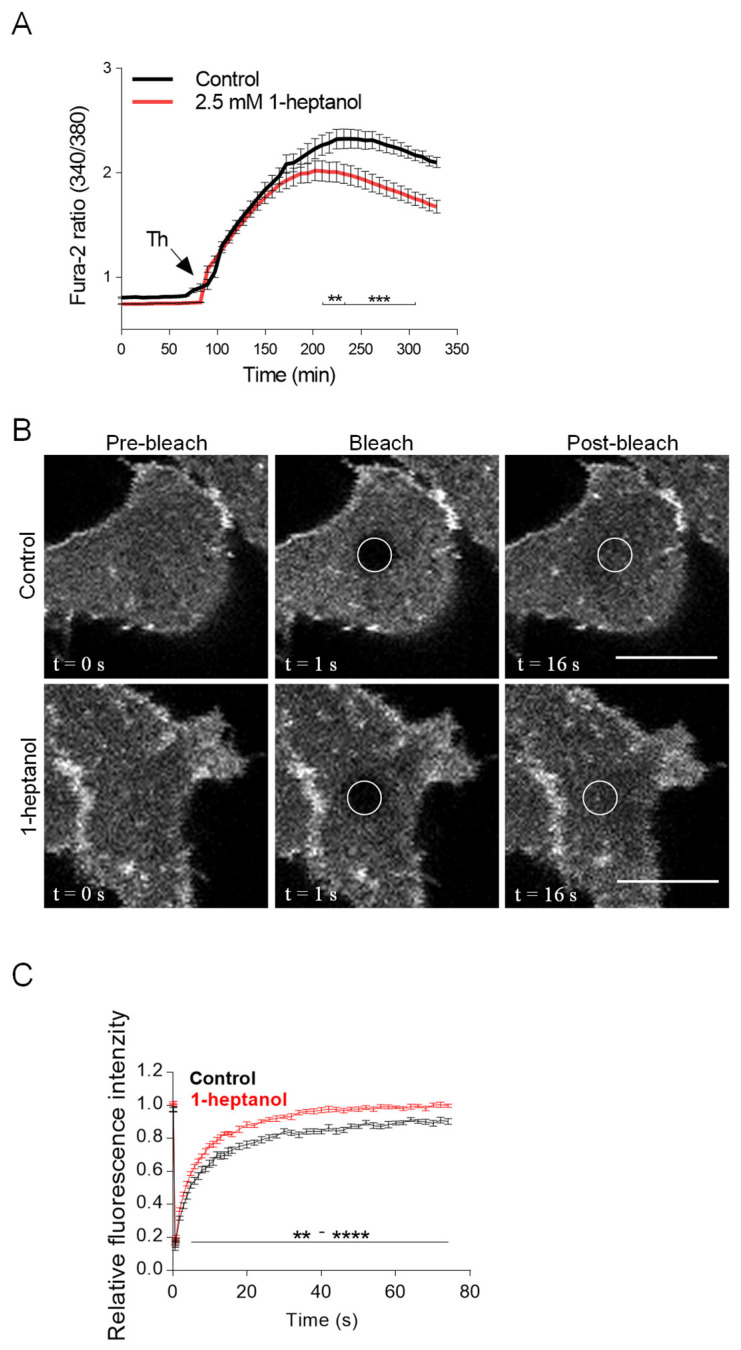
1-Heptanol decreased calcium mobilization but increased ORAI1-mCherry lateral diffusion in HEK293 cells. (**A**) Intracellular calcium mobilization in HEK293 cells. The cells were loaded with Fura 2-AM alone (Control; black line; *n* = 10) or with Fura 2-AM and 2.5 mM 1-heptanol (red line; *n* = 12) for 15 min. The cells were then activated by adding 1 µM thapsigargin (Th, arrow), and calcium response was monitored by measuring fluorescence ratios. The values indicate means ± SEM. The bars under the curves denote statistically significant differences between control and 1-heptanol-treated cells at the indicated time intervals; ** *p* < 0.01; *** *p* < 0.001. (**B**) 1-Heptanol enhances the membrane mobility of ORAI1 as determined by FRAP. HEK293 cells transfected with ORAI1-mCherry were exposed to the vehicle (BSS-BSA; Control) or 2.5 mM 1-heptanol for 10 min. Representative fluorescence image sequences are from cells at the indicated time points before photobleaching (pre-bleach), at photobleaching (bleach; the circled areas), or after photobleaching (post-bleach; the circled areas). Bars indicate 10 µm. (**C**) Quantification of the recovery into the photobleached areas as shown in (**B**). Relative fluorescence intensity was calculated at each time point by dividing the fluorescence in the bleached region by the fluorescence in the corresponding unbleached region. The background was subtracted from all signals. Values in (**C**) indicate the means ± SEM of two independent experiments for the control cells (*n* = 14) and 1-heptanol-treated cells (*n* = 15). Statistical differences between groups in the range ** *p* < 0.01–**** *p* < 0.0001 are indicated in (**C**).

We hypothesized that 1-heptanol-mediated changes in the mobility of plasma membrane components could cause changes in the lateral diffusion of ORAI1 channels and subsequently alter the STIM1/ORAI1 interaction dynamics, which could explain the reduced calcium response in 1-heptanol-treated cells. To determine the changes in ORAI1 mobility and STIM1/ORAI1 coupling, we used HEK293 cells, which can be efficiently transfected with plasmid vectors and show a decrease in thapsigargin-mediated calcium mobilization in the presence of 1-heptanol (Figure 7A). We found that the mobility of ORAI1-mCherry was increased in the presence of 1-heptanol (Figure 7B,C). Quantification of the normalized curves yielded an ORAI1-mCherry diffusion coefficient of 0.082 ± 0.022 µm^2^ s^−1^ in control cells, whereas in 1-heptanol-treated cells it was 0.076 ± 0.022 µm^2^ s^−1^; the dissociation rate (k^off^) was 0.11 ± 0.03 s^−1^ in control cells and 0.15 ± 0.03 s^−1^ in cells exposed to 1-heptanol. The immobile fraction of ORAI1-mCherry in control cells was 7.93 ± 3.91% and 1.4 ± 1.78% in 1-heptanol-treated cells, indicating that ORAI1-mCherry is almost free to diffuse in these cells and that 1-heptanol enhances the diffusion.

**Figure 8:**
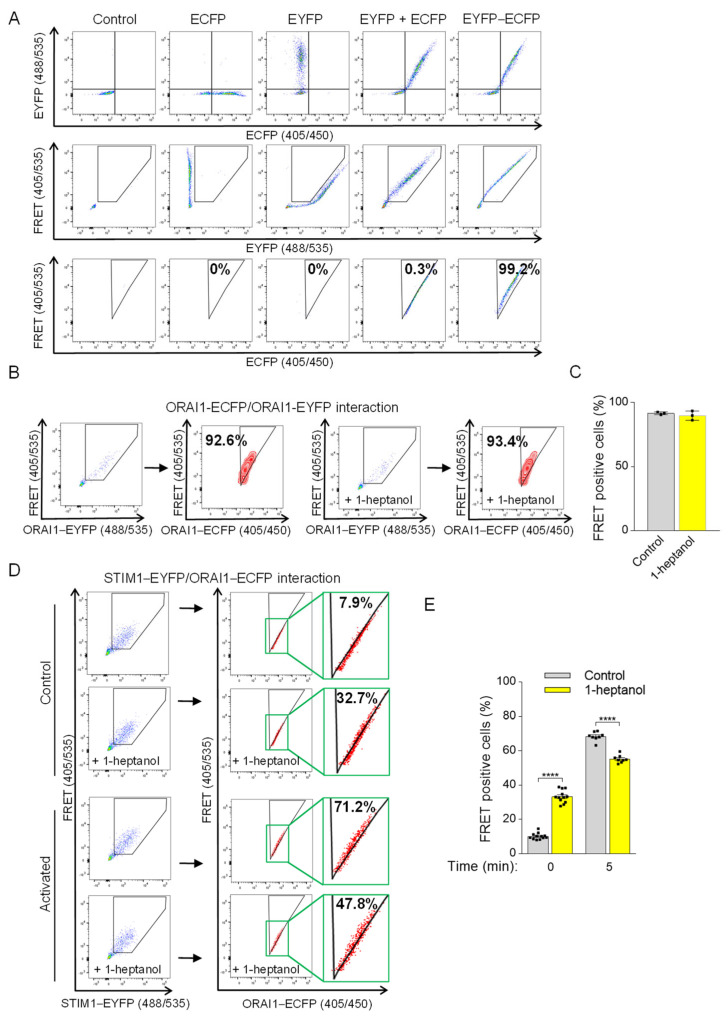
1-Heptanol-induced changes interfere with ORAI1 and STIM1 interactions. (**A**) The flow-FRET method in HEK293 cells for determining the sensitized FRET emission between EYFP and ECFP pairs. Co-expression of EYFP and ECFP determined a negative FRET signal compared to cells expressing the EYFP–ECFP construct, which corresponds to FRET-positive cells displayed in the triangular gate (FRET-positive gate) as a percentage of transfected cells excited at 405 nm with simultaneous photon collection of ECFP and EYFP emission (shown in third line). The Section 2 and Section 3 provide more details on the method and gating strategy. (**B**) ORAI1 homomultimers determined by flow-FRET in untreated (*n* = 3) or 2.5 mM 1-heptanol-treated (+1-heptanol; *n* = 3) HEK293 cells transfected with ORAI1–ECFP and ORAI1-EYFP. (**C**) Quantitative analysis of cells in the FRET-positive gate as shown in B. (**D**) The interaction of STIM1–EYFP with ORAI1–ECFP was determined in cells pretreated with vehicle (BSS-BSA, *n* = 12) or 2.5 mM 1-heptanol (+1-heptanol; *n* = 8) using flow-FRET. Then, the transfected cells were activated with thapsigargin at a final concentration of 1 µM; 5 min later, cells in the FRET-positive gate were measured. The rectangles bordered by the green box show the enlarged area in the triangular gate. (**E**) Quantitative analysis of cells in the FRET-positive gate as shown in (**D**). Representative flow cytometry profiles are presented. Values indicate means ± SEM calculated from *n*, which show the numbers of biological replicates; **** *p* < 0.0001.

To measure 1-heptanol-induced changes in STIM1/ORAI1 coupling, we introduced a flow cytometry-based FRET assay according to Banning et al. [48]. The assay measures the sensitized emission of the acceptor in living cells. In our study, we used ECFP as the photon donor and EYFP as the acceptor (Figure 8A). The expression of fluorescent proteins expressed in HEK293 cells (ECFP alone, EYFP alone, ECFP together with EYFP, or EYFP conjugated to ECFP (ECFP-EYFP)) was evaluated by flow cytometry (Figure 8A, top line). The gating strategy to separate the 488 nm laser beam-dependent basal EYFP signal from the ECFP/EYFP co-expression signal (shown in a pentagonal gate) is shown in the middle row of Figure 8A. Cells co-expressing ECFP and EYFP were used to determine the threshold of random energy transfer between the ECFP and EYFP pair when excited with a 405 nm laser (FRET negative signal; Figure 8A, bottom line). The triangular gate was positioned to exclude almost all cells from this gate (remaining ~0.3%). The fluorescence of the conjugated ECFP-EYFP construct in the positive FRET channel (FRET 405/535) was shifted to the triangular gate (~99.2%), as the excitation of ECFP caused energy transfer to EYFP and increased the 405 nm excitation-mediated emission in the 535 nm channel (Figure 8A, bottom line). This setup was used in the following studies (Figure 8B–E). Because 1-heptanol has been shown to interfere with luciferase activity [21], we first tested the FRET between ORAI1-ECFP and ORAI1-EYFP dimers in the absence or presence of 2.5 mM 1-heptanol to rule out its interference with photon-dependent energy transfer. We found that the FRET measurement between ORAI1-ECFP and ORAI1-EYFP was unaffected in cells exposed to 2.5 mM 1-heptanol for 10 min (89.8 ± 2.1) compared to untreated cells (91.6 ± 0.6; Figure 8B,C). Coupling between ORAI1-ECFP and STIM1-EYFP in transfected HEK293 cells was measured in resting cells in the presence or absence of 2.5 mM 1-heptanol. Interestingly, cells exposed to 1-heptanol for 10 min exhibited an increased population of cells in the FRET-positive gate (33.3 ± 1.1) compared to untreated cells (10.1 ± 0.5; Figure 8D,E). These data suggest that 1-heptanol induced changes in the ER membrane and in the translocation of STIM1 to the vicinity of the plasma membrane. This coupling was not accompanied by increased Ca^2+^ levels in resting HEK293 cells exposed to 2.5 mM 1-heptanol (Figure 7A). Next, we added thapsigargin to the cells and measured coupling after 5 min. In thapsigargin-activated cells not exposed to 1-heptanol, 68.4 ± 0.9% of cells were shifted into the FRET-positive gate compared to 55.2 ± 0.8% of 1-heptanol-treated cells (Figure 8D,E). These data suggest that 1-heptanol directly or indirectly interferes with the assembly of CRAC channels.

### 3.6. Impaired Cytokine Production in 1-Heptanol-Treated BMMCs

**Figure 9:**
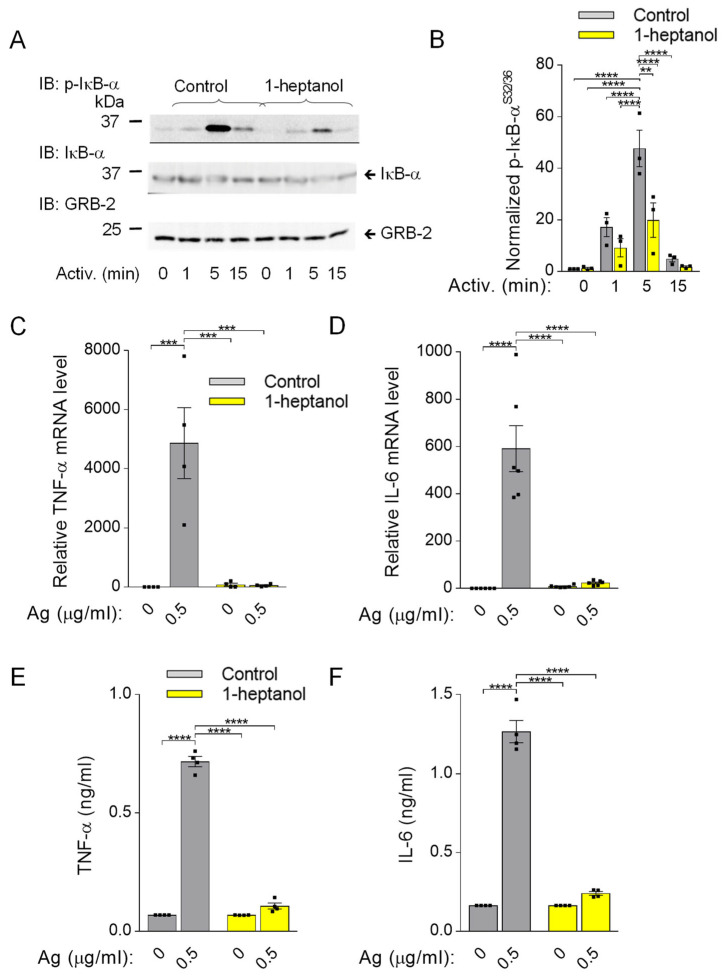
Impaired IκB-α phosphorylation and cellular TNF-α expression in 1-heptanol-treated BMMCs. (**A**) IκB-α phosphorylation (p-IκB-α) and expression of IkB-α and GRB-2 were determined by SDS-PAGE size-fractionation and IB of lysates from BMMCs treated with 2.5 mM 1-heptanol or vehicle (Control) and activated for the indicated time intervals with antigen (TNP-BSA; 0.5 µg/mL; *n* = 3). Representative immunoblots developed with the corresponding antibodies are shown. (**B**) The results from the quantification of data as in **A** normalized to signals in non-activated cells and loading control proteins. (**C**,**D**) RT-PCR quantification of TNF-α mRNA ((**C**); *n* = 4) and IL-6 ((**D**); *n* = 6) in non-activated or antigen-activated (Ag; 0.5 µg/mL; 1h) BMMCs pretreated for 15 min with 2.5 mM 1-heptanol or vehicle (Control). (**E**,**F**) The levels of TNF-α (**E**) and IL-6 (**F**) released into the supernatant of IgE-sensitized BMMCs pretreated with vehicle (Control; *n* = 4) or 2.5 mM 1-heptanol (*n* = 4) and non-activated or activated with antigen (Ag) for 4 h. Values indicate means ± SEM calculated from *n*, which show the numbers of biological replicates; ** *p* < 0.01, *** *p* < 0.001, **** *p* < 0.0001.

Because phosphorylation of IκB-α is a prerequisite for FcεRI-mediated cytokine production [33], we assessed this signaling pathway in BMMCs pretreated with 2.5 mM 1-heptanol. In contrast to the phosphorylation cascade in the SYK/LAT1/PLCγ1 signaling pathway in antigen-activated BMMCs (Figure 3D–I), IκB-α phosphorylation was significantly reduced in 1-heptanol-treated cells as compared to control cells at 1 and 5 min after antigen triggering (Figure 9A,B). The densitometric evaluation of the phosphorylated IκB-α was normalized to GRB2, as the levels of IκB-α are reduced upon activation [32]. The observed decrease in IκB-α phosphorylation in antigen-activated cells was associated with impaired expression of the *TNFa* and *IL6* gene in 1-heptanol-treated cells (Figure 9C,D). As expected, the production of TNF-α and IL-6 at the protein level, as measured by bead-based immunoassay, was suppressed entirely in 1-heptanol-treated cells activated with the indicated concentrations of antigen (Figure 9E,F). These data are in accordance with a previous study showing the effects of n-alkanols on IFN-γ production in peripheral blood T lymphocytes [27].

### 3.7. Short-Term Exposure to 1-Heptanol Suppresses Production of ROS and LTC4

**Figure 10:**
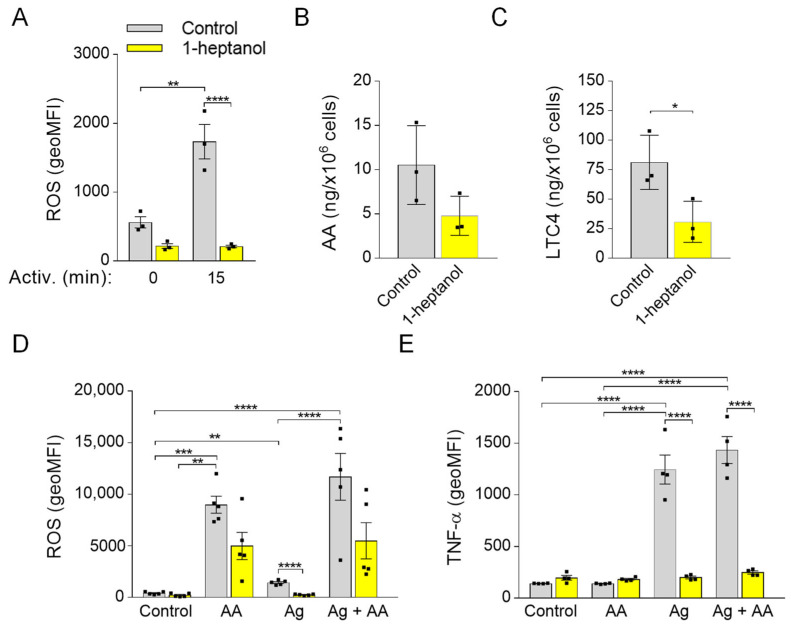
Reduced production of ROS and LTC4 in 1-heptanol-treated cells and the effect of ROS restoration by arachidonic acid on TNF-α production in BMMCs. (**A**) IgE-sensitized and H2DCFDA-loaded BMMCs were incubated for 15 min in the presence of a vehicle (BSS-BSA; Control; *n* = 3) or 2.5 mM 1-heptanol (*n* = 3). BMMCs were then activated with antigen (Ag; 0.5 µg/mL). Fifteen min later, the fluorescence signal was quantified by flow cytometry. (**B**,**C**) UPLC MS/MS analysis of arachidonic acid (AA; B) and LTC4 (**C**) from supernatants of untreated (Control; *n* = 3) or 1-heptanol-treated (*n* = 3) antigen-activated BMMCs. (**D**) IgE-sensitized and H2DCFDA-loaded BMMCs were prepared as in A. BMMCs were then untreated (Control; *n* = 5) or treated with arachidonic acid (*n* = 5), antigen (0.5 µg/mL; *n* = 5), or antigen in combination with arachidonic acid (*n* = 5). The fluorescence signal was quantified as above. (**E**) Analysis of cellular TNF-α production by flow cytometry in non-activated IgE-sensitized BMMCs (Control; *n* = 4), cells exposed for 1.5 h to arachidonic acid (*n* = 4), antigen alone (*n* = 4) or antigen and arachidonic acid (*n* = 4), and concurrently in the presence or absence of 2.5 mM 1-heptanol. Values indicate means ± SEM calculated from *n*, which show the numbers of biological replicates; * *p* < 0.05, ** *p* < 0.01, *** *p* < 0.001, **** *p* < 0.0001.

Previously, we found that 0.5% ethanol partially but significantly decreased the production of ROS in antigen-activated BMMCs and that MβCD potentiated the inhibitory effect. Interestingly, pretreatment of the cells with sMβCD restored the ROS levels and FcεRI signaling in ethanol-treated cells [50]. In this study, we examined the effect of 1-heptanol on ROS production and found that 1-heptanol had a substantial inhibitory effect on ROS production in antigen-activated cells (Figure 10A). In antigen-activated human and mouse mast cells, 5-lipoxygenase is responsible for most ROS production [72]. Therefore, we examined the levels of arachidonic acid, a 5-lipoxygenase substrate, released from the plasma membrane by the activity of phospholipases [73]. We found that arachidonic acid was downregulated (*p* = 0.1) in 1-heptanol-treated cells (Figure 10B) but that LTC4, which depends on the activity of 5-lipoxygenase and LTC4 synthase [73], was decreased (Figure 10C). Next, we examined the effect of cells treated with external arachidonic acid. To this end, we pre-incubated cells with or without arachidonic acid in the presence or absence of 1-heptanol. We found that externally added arachidonic acid induced ROS production and partially restored ROS production in 1-heptanol-treated and FcεRI-activated cells (Figure 10D), but did not reverse the inhibitory effect of 1-heptanol on TNF-α production (Figure 10E).

### 3.8. 1-Heptanol-Mediated Plasma Membrane Hyperfluidization Causes Increased HSP70 Expression

**Figure 11:**
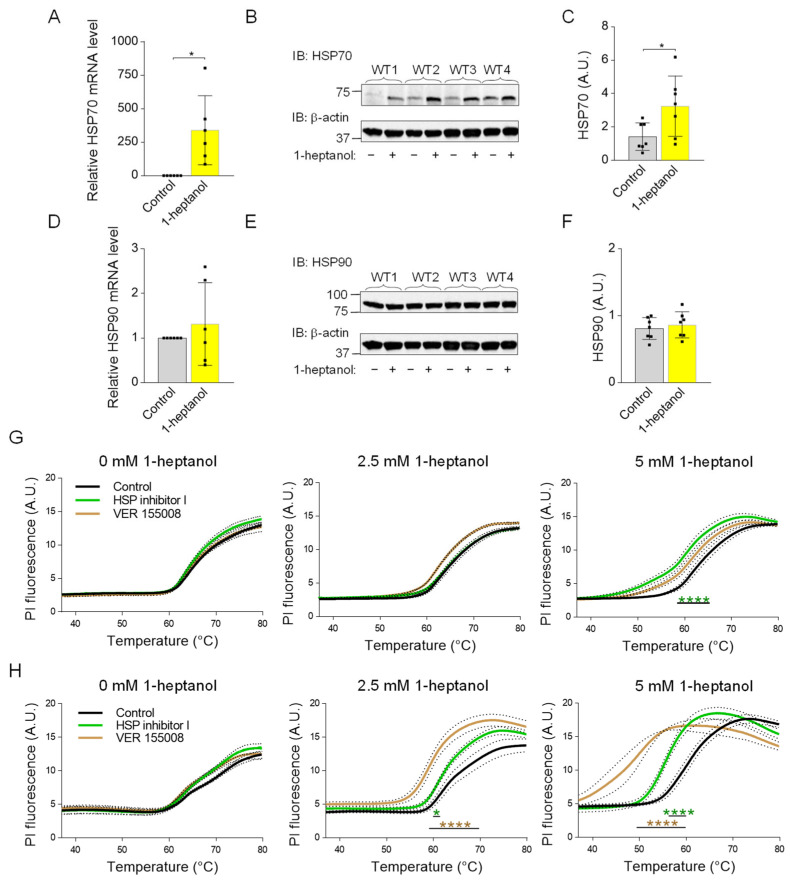
Enhanced expression of HSP70 in 1-heptanol-treated BMMCs and the thermal stability of the plasma membrane in the presence of 1-heptanol and inhibitors affecting HSP70 function or expression. (**A**) RT-PCR quantification of HSP70 mRNA in non-activated (*n* = 6) BMMCs pretreated for 15 min with 2.5 mM 1-heptanol or vehicle (Control). (**B**) HSP70 expression levels in BMMCs untreated (−) or treated (+) with 2.5 mM 1-heptanol for 75 min. (**C**) Quantitative analysis of the HSP70 levels normalized to β-actin expression as in B. Values are means ± SEM calculated from seven biological replicates. (**D**) RT-PCR quantification of HSP90 mRNA in non-activated (*n* = 6) BMMCs pretreated for 15 min with 2.5 mM 1-heptanol or vehicle (Control). (**E**) Expression levels of HSP90 in control BMMCs and BMMCs treated with 2.5 mM 1-heptanol for 75 min. (**F**) Quantitative analyses of the HSP90 levels normalized to β-actin expression as in E. Values indicate means ± SEM calculated from seven biological replicates. * *p* < 0.05. (**G**) Membrane thermal stability analysis of BMMCs pretreated overnight with vehicle (DMSO; Control; *n* = 6), HSP inhibitor I (*n* = 3), or VER 155008 (*n* = 3) in the absence (0 mM) or presence of 2.5 mM or 5 mM 1-heptanol. (**H**) Membrane thermal stability analysis of RBL-2H3 cells pretreated overnight with vehicle (DMSO; Control; *n* = 6), HSP inhibitor I (40 μM, *n* = 3), or VER 155008 (25 μM, *n* = 3) in the absence (0 mM) or presence of 2.5 mM or 5 mM 1-heptanol. Cells in (**G**,**H**) were exposed to the indicated concentrations of 1-heptanol and PI. The cells were plated in 96-well plates for RT-PCR and incubated for 15 min at 37 °C, followed by PI fluorescence measurement at a slowly increasing temperature in the RT-PCR instrument. Values indicate means ± SEM calculated from *n*, which show the numbers of biological replicates. Intergroup differences between control and heptanol-treated cells were examined by two-way ANOVA; * *p* < 0.05, **** *p* < 0.0001.

The membrane fluidizers 1-heptanol and benzyl alcohol mimic the heat stress-dependent induction of HSP70 expression [74]. Because HSP70 is a potent inhibitor of pro-inflammatory cytokine expression [75], we tested HSP70 expression in BMMCs treated with 1-heptanol. We found that cells incubated with 2.5 mM 1-heptanol exhibited increased expression of HSP70 when compared to control cells treated with the vehicle at both the mRNA (Figure 11A) and protein (Figure 11B,C) levels. Previous studies have shown that benzyl alcohol specifically induces the expression of HSP70 but not HSP90 [76]. Consistent with this finding, we found that in contrast to HSP70, HSP90 levels are unchanged in 1-heptanol-treated cells (Figure 11D–F). The data suggest that 1-heptanol triggers the heat stress response and explicitly induces the expression of HSP70 but not HSP90.

### 3.9. 1-Heptanol-Potentiated Plasma Membrane Permeability, Measured by Membrane Thermal Integrity Assay Is Further Enhanced by HSP70 Inhibitors

To determine the protective role of HSP70 in the presence of 1-heptanol, we performed a membrane thermal integrity assay in BMMCs and RBL-2H3 cells pre-incubated overnight with two HSP70 inhibitors: HSP nonspecific inhibitor I (KNK437), which inhibits expression of a variety of HSPs [77], and HSP70 specific inhibitor VER 155008, which competes with ATP for the nucleotide-binding pocket site, resulting in impaired HSP70 chaperone activity [78]. We found that the integrity of the BMMC plasma membrane was resistant to both inhibitors in the absence or presence of 2.5 mM 1-heptanol. A decreased membrane integrity was observed in cells pretreated with HSP inhibitor I and 5 mM 1-heptanol (Figure 11G). In contrast, an increased plasma membrane permeability to PI was observed in RBL-2H3 cells pretreated with both inhibitors and incubated with 2.5 mM 1-heptanol, and especially with 5 mM 1-heptanol, compared to control cells treated overnight with vehicle (DMSO) only. Interestingly, RBL-2H3 cells were more sensitive to the HSP-70 specific inhibitor VER 155008 than to the HSP70 nonspecific HSP inhibitor I (Figure 11H). Thus, the data show a cell type-specific effect of HSP70 on the thermal membrane integrity in the presence of 1-heptanol.

## 4. Discussion

Aliphatic chain alcohols are modifiers of plasma membrane properties due to their long hydrophobic acyl groups [18,23]. Cholesterol oxidases interact only superficially with Lo-phase organized cholesterol [79] unless amphipathic molecules, such as aliphatic alcohols, displace the plasma membrane cholesterol from its phospholipid complexes and facilitate its exposure to their enzymatic activity [18,19]. This process, termed cholesterol activation, is associated with increased membrane fluidity, and is followed by segregating proteins and lipids with the Lo-like phase preference into micron-sized aggregates [31]. In this study, we examined the effect of a volatile aliphatic C_7_-chain alcohol, 1-heptanol, on mast cell properties and signaling in vitro.

Our data indicate that the 1-heptanol-mediated increase in FcεRI lateral mobility does not affect early signaling events in antigen-activated mast cells such as phosphorylation of the FcεRI-β chain and SYK/LAT1/PLCγ1 signaling axis. These data are opposite to our recent studies showing that the ursolic acid-mediated decrease in FcεRI lateral mobility was associated with increased phosphorylation of the FcεRI-β chain, SYK, and PLCγ1 [30]. Treatment with both 1-heptanol (this study) and ursolic acid [30] decreased degranulation and calcium mobilization, but differed in their effects on TNF-α production, which was increased in ursolic acid-treated cells but inhibited in 1-heptanol-treated cells. The output of early FcεRI signaling events may differ depending on the type of alcohol used in the assay. Previously, we have shown that antigen-activated BMMCs pretreated with 0.5% ethanol exhibited decreased phosphorylation of the FcεRI-β chain, SYK, LAT1, PLCy1, and ERK, followed by decreased degranulation, calcium mobilization, cytokine secretion, and ROS production [50]. MβCD-mediated cholesterol depletion potentiated the effect of 0.5% ethanol on degranulation, calcium response, and ROS production [50]. The combined data indicate that changes in the initial steps of FcεRI signaling may differ in ethanol- and 1-heptanol-treated cells. However, the effector functions, such as degranulation and cytokine production, may be similarly affected. We found that FcεRI internalization is delayed in the presence of 1-heptanol, which likely prolongs FcεRI signaling at the plasma membrane and could offset the inhibitory effect of 1-heptanol on phosphorylation of the FcεRI-β chain, SYK, LAT1, and PLCγ1. The lateral mobility of FcεRI seems to have an impact on TNF-α production, as BMMCs exposed to ursolic acid exhibited frozen mobility of FcεRI and increased production of cellular TNF-α [30], whereas 1-heptanol, inducing increased FcεRI diffusion, inhibited the TNF-α production. It has been shown that cells treated with benzyl alcohol or 1-heptanol exhibit a heat shock-like response by expression of HSP70, comparable in extent to the response to thermal shift up to 42 °C [74,76]. The authors of this finding proposed that the increased membrane fluidity caused by the fluidizers mimics the effect of the increased temperature. It should be mentioned that the HSP70 protein is a potent inhibitor of IκB-α phosphorylation, crucial for translocating NF-κB into the nuclei of activated cells [75]. Our data show that 1-heptanol increases HSP70 levels in non-activated BMMCs and decreases the phosphorylation of IκB-α in the cells after antigen activation. This results in reduced transcription of cytokine genes.

In BMMCs exposed to heat shock and then stimulated with antigen, the cells exhibited reduced β-glucuronidase release, calcium mobilization, and production of the cytokines TNF-α and IL-6 [80,81]. While these data are consistent with our study, it has been shown that heat shock inhibits SYK phosphorylation in antigen-activated BMMCs [80]; thus, the effect of 1-heptanol and heat stress overlap only partially. To examine the effect of 1-heptanol on thermal membrane stability, we developed an assay in which cells in wells of RT-PCR plates are mixed with 1-heptanol and PI; the RT-PCR instrument monitors their fluorescence in the wells at increasing temperatures. Using this method, we showed that 1-heptanol increases temperature instability depending on the 1-heptanol concentrations. This instability was cell type-specific; BMMCs were more resistant to increasing temperature in the presence of 1-heptanol than RBL-2H3 and human ROSA cells. The literature data suggest that HSPs, including the well-studied HSP70, are inserted into the cell membranes, stabilizing them and serving as a primary sensor for thermal stress [82]. In our experiments, we found that the membrane permeability to PI was increased in BMMCs and in RBL cells treated with HSP70 inhibitors and simultaneously exposed to 1-heptanol and stress conditions (heat stress), supporting the concept of an important role of HSP70 in maintaining membrane integrity.

Antigen-activated BMMCs treated with 1-heptanol exhibited decreased calcium mobilization. However, because phosphorylation of the FcεRI/SYK/LAT1/PLCγ1 axis in 1-heptanol-treated cells showed normal tyrosine phosphorylation, it is likely that 1-heptanol reduced the CRAC channel activity. This possibility is supported by impaired Ca^2+^ mobilization in 1-heptanol-treated mast cells activated by thapsigargin and 1-heptanol-mediated changes in lateral mobility of fluorescently labeled CRAC channel subunit (ORAI1-mCherry) in HEK293 cells. Calcium ions serve as an intracellular second messenger and are involved in signal transduction in many cellular pathways, leading to NF-κB and NFAT activation followed by cytokine expression [83], ROS production [84], stimulation of 5-lipoxygenase activity [85], and degranulation, which depends on microtubule reorganization [70]. Our data suggest that the decreased Ca^2+^ mobilization in 1-heptanol-treated mast cells contributes to impaired mast cell effector functions. Critical components of SOCE are STIM1 and ORAI1, which interact at the plasma membrane-ER contact site [63,64]. Antigen-activated mast cells deficient in STIM1 show impaired Ca^2+^ mobilization, NF-κB and NFAT activation, cytokine production, and degranulation [32]. To analyze the STIM1-ORAI1 interactions we used HEK293 cells, which exhibited reduced calcium mobilization induced by thapsigargin after 1-heptanol treatment. We found that the basal interaction in 1-heptanol-treated HEK293 cells overexpressing STIM-EYFP and ORAI1-ECFP as measured by flow-FRET was increased compared to untreated cells. However, after thapsigargin activation the coupling levels in 1-heptanol-treated cells were significantly lower than in control cells. These data suggest a role of 1-heptanol-mediated hyperfluidization in this process. Indeed, it has been shown that Lo-like domains are essential for SOCE [12,13] and that major proteins involved in this process, namely, STIM1 and ORAI1, possess functional cholesterol-binding domains [15,16]. Consistent with our results, HEK293 cells and RBL cells treated with MβCD to reduce plasma membrane cholesterol showed decreased calcium mobilization in thapsigargin-activated cells [12,14]. These data are supported by studies showing that elevated cholesterol levels in sMβCD-treated BMMCs exhibited increased calcium mobilization in antigen-activated cells [50]. However, increased calcium mobilization was observed after thapsigargin exposure in another study using RBL cells and HEK293 with cholesterol depletion by cholesterol oxidase [15]. Thus, the studies targeting cholesterol depletion in membranes by different approaches show conflicting data on calcium mobilization. Our findings support previous studies showing that the coupling between STIM1-mRFP and AcGFP-ORAI1, measured by FRET was decreased in thapsigargin-activated RBL cells exposed to MβCD [12]. Similarly, the STIM1-ORAI1 interaction measured by double-sided immunoprecipitation in human platelets showed that MβCD pretreatment decreased STIM1-ORAI1 coupling and sMβCD restored the STIM1-ORAI1 interaction [13]. These data are consistent with our flow-FRET results as well. The cholesterol-binding domain of ORAI1 is located in the extended transmembrane ORAI1 N-terminal (ETON) region involved in ORAI1 interaction with calmodulin, CRACR2A, and STIM1 [15]. The ETON region is close to the N-terminal part of ORAI1, which is responsible for the cAMP-dependent feedback loop in the process termed Ca^2+^-dependent inactivation [86]. In addition, STIM1 and ORAI1 proteins exhibit differential sensitivity to phosphatidylinositol 4,5-bisphosphate in the specific membrane domains that are essential for SOCE [12]. This complexity may explain why the 1-heptanol-mediated membrane perturbations showed increased coupling of ORAI1 and STIM1 in resting cells without an increase in basal Ca^2+^ levels.

The combined data suggest that cytokine production in immune cells is regulated by a variety of pathways, including calcium influx, which precedes NF-κB and NFAT activation [32,83], heat stress [80,81], and SAPK/JNK phosphorylation playing a central role in stress signaling pathways [66,87]. Interestingly, in MAPK kinase 2 (MEKK2)-deficient mast cells derived from embryonic stem cells, the phosphorylation of the MEKK2 downstream target SAPK/JNK was impaired upon antigen activation, while the production of IL-4, IL-6, and TNF-α was inhibited; at the same time, phosphorylation of p38 and ERK was not affected [87]. This corresponds to our data on BMMCs showing that 1-heptanol inhibited cytokine production without any effect on p38 and ERK phosphorylation, but selectively decreased SAPK/JNK phosphorylation in antigen-activated cells.

The role of ROS in mast cell-mediated cytokine production is controversial. In the human mast cell line (HMC)-I, ROS generated by phorbol myristate acetate (PMA) stimulated the expression of *IL8* and *TNFa* genes [88]. However, in another study using BMMCs and human mast cells cultured from CD34+ peripheral blood mononuclear cells, the most potent ROS producer was 5-lipoxygenase, an enzyme that converts arachidonic acid to LTA4, and showed complete inhibition of FcεRI-mediated ROS production without effect on degranulation or cytokine expression [72]. Because both ROS-producing pathways, 5-lipoxygenase and PMA-stimulated NADPH oxidase, are associated with the cell membrane, we tested the possible role of 1-heptanol on the ROS production. We found that its production was completely inhibited in 1-heptanol-treated and antigen-activated cells. This inhibition was reversed when arachidonic acid was added, suggesting that 5-lipoxygenase is partially functional, as was confirmed by examining LTC4 levels in 1-heptanol-treated cells.

## 5. Conclusions

**Figure 12:**
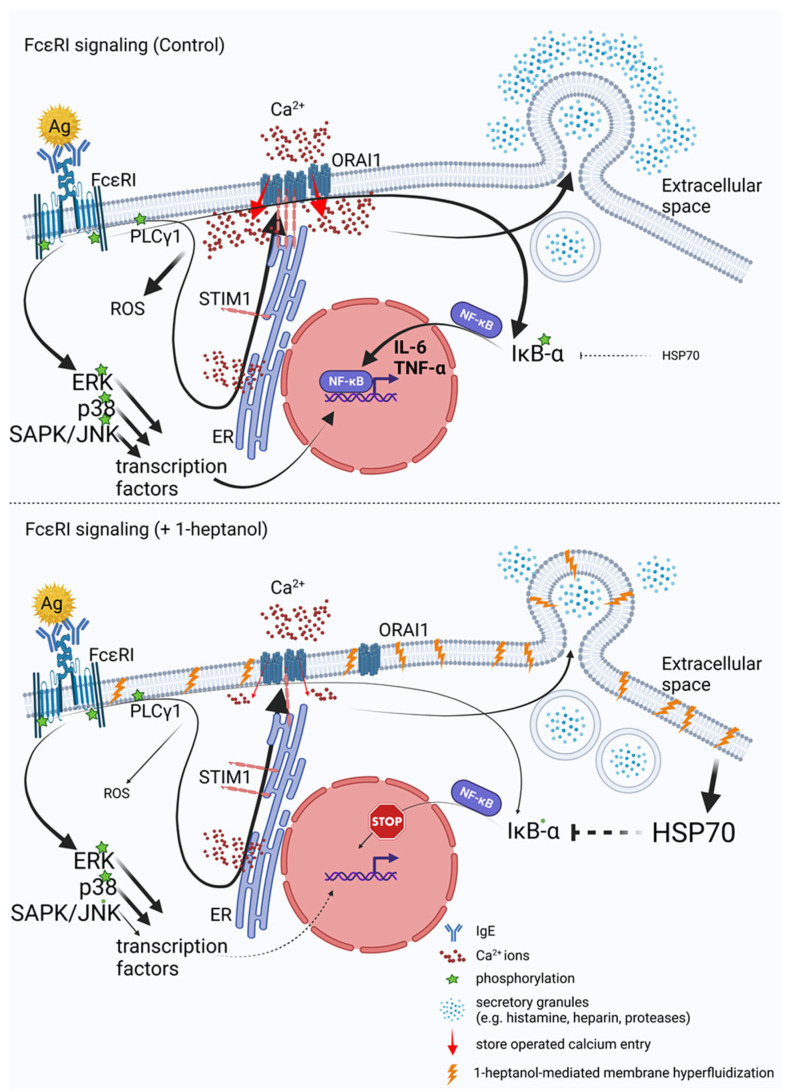
Model of a possible mechanism by which 1-heptanol inhibits FcεRI-mediated mast cell activation. FcεRI signaling in control and 1-heptanol-treated cells are shown on the top and bottom, respectively. The initial steps in FcεRI signaling, including FcεRI and PLCγ1 phosphorylation and release of Ca^2+^ from the endoplasmic reticulum (ER) stores, are similar in control and 1-heptanol-treated cells. However, ORAI1-STIM1 coupling is decreased in 1-heptanol-treated cells activated with antigen, suggesting that the resulting membrane hyperfluidization disrupts CRAC channels. The reduced influx of Ca^2+^ ions from extracellular space is associated with impaired degranulation and ROS production. In addition, exposure to 1-heptanol leads to overexpression of HSP70 and inhibition of IκB-α and SAPK/JNK phosphorylation, contributing to the inhibition of cytokine production.

This study shows that 1-heptanol increases membrane fluidity, as measured by FRAP, of FITC-IgE-labeled FcεRI complexes and ORAI1-mCherry. Increased lateral mobility of FcεRI and reduced FceRI internalization due to 1-heptanol do not interfere with the initial steps in antigen-activated mast cells, as reflected by enhanced phosphorylation of proteins of early signaling events, including FcεRI-β chain, SYK, LAT1, and PLCγ1. Nevertheless, calcium response, degranulation, ROS production, and TNF-α levels were impaired in 1-heptanol-treated and FcεRI-activated cells. Further analysis showed that 1-heptanol-mediated membrane hyperfluidization altered the dynamics of STIM1-ORAI1 coupling in resting and activated cells, as determined by flow-FRET, resulting in reduced calcium uptake. These events may be associated with the enhanced lateral mobility of ORAI1. Membrane hyperfluidization mediated by 1-heptanol enhanced the sensitivity of cells to increased temperature and resulted in enhanced production of HSP70, which could contribute to the inhibition of cytokine expression. These findings are summarized in Figure 12. The combined data prove that 1-heptanol inhibits mast cell effector functions; this should be considered when 1-heptanol is used in the food and fragrance industries.

## Data Availability

All data are provided herein.
